# Effectiveness of a mHealth Lifestyle Program With Telephone Support (TXT2BFiT) to Prevent Unhealthy Weight Gain in Young Adults: Randomized Controlled Trial

**DOI:** 10.2196/mhealth.4530

**Published:** 2015-06-15

**Authors:** Stephanie R Partridge, Kevin McGeechan, Lana Hebden, Kate Balestracci, Annette TY Wong, Elizabeth Denney-Wilson, Mark F Harris, Philayrath Phongsavan, Adrian Bauman, Margaret Allman-Farinelli

**Affiliations:** ^1^ School of Molecular Bioscience Charles Perkins Centre University of Sydney Sydney Australia; ^2^ Sydney School of Public Health Charles Perkins Centre University of Sydney Sydney Australia; ^3^ Faculty of Health University of Technology Sydney Sydney Australia; ^4^ Centre for Primary Health Care and Equity University of New South Wales Sydney Australia

**Keywords:** young adults, weight gain prevention, lifestyle behavior, mHealth

## Abstract

**Background:**

Weight gained in young adulthood often persists throughout later life with associated chronic disease risk. Despite this, current population prevention strategies are not specifically designed for young adults.

**Objective:**

We designed and assessed the efficacy of an mHealth prevention program, TXT2BFiT, in preventing excess weight gain and improving dietary and physical activity behaviors in young adults at increased risk of obesity and unhealthy lifestyle choices.

**Methods:**

A two-arm, parallel-group randomized controlled trial was conducted. Subjects and analyzing researchers were blinded. A total of 250 18- to 35-year-olds with a high risk of weight gain, a body mass index (BMI) of 23.0 to 24.9 kg/m^2^ with at least 2 kg of weight gain in the previous 12 months, or a BMI of 25.0 to 31.9 kg/m^2^ were randomized to the intervention or control group. In the 12-week intervention period, the intervention group received 8 text messages weekly based on the transtheoretical model of behavior change, 1 email weekly, 5 personalized coaching calls, a diet booklet, and access to resources and mobile phone apps on a website. Control group participants received only 4 text messages and printed dietary and physical activity guidelines. Measured body weight and height were collected at baseline and at 12 weeks. Outcomes were assessed via online surveys at baseline and at 12 weeks, including self-reported weight and dietary and physical activity measures.

**Results:**

A total of 214 participants—110 intervention and 104 control—completed the 12-week intervention period. A total of 10 participants out of 250 (4.0%)—10 intervention and 0 control—dropped out, and 26 participants (10.4%)—5 intervention and 21 control—did not complete postintervention online surveys. Adherence to coaching calls and delivery of text messages was over 90%. At 12 weeks, the intervention group were 2.2 kg (95% CI 0.8-3.6) lighter than controls (*P*=.005). Intervention participants consumed more vegetables (*P*=.009), fewer sugary soft drinks (*P*=.002), and fewer energy-dense takeout meals (*P*=.001) compared to controls. They also increased their total physical activity by 252.5 MET-minutes (95% CI 1.2-503.8, *P*=.05) and total physical activity by 1.3 days (95% CI 0.5-2.2, *P*=.003) compared to controls.

**Conclusions:**

The TXT2BFiT low-intensity intervention was successful in preventing weight gain with modest weight loss and improvement in lifestyle behaviors among overweight young adults. The short-term success of the 12-week intervention period shows potential. Maintenance of the behavior change will be monitored at 9 months.

**Trial Registration:**

Trial Registration: The Australian New Zealand Clinical Trials Registry ACTRN12612000924853; https://www.anzctr.org.au/Trial/Registration/TrialReview.aspx?ACTRN=12612000924853 (Archived by WebCite at http://www.webcitation.org/6Z6w9LlS9).

## Introduction

More than 35% of adults globally are overweight or obese, and in developed countries the peak prevalence of obesity is moving to younger ages [1]. For example, younger Americans and Australians are gaining more weight than any other adult age group [2-4]. As body mass index (BMI) exceeds 23 kg/m^2^, risks of cardiovascular disease, certain cancers, diabetes, osteoarthritis, and chronic kidney disease increase [1]. The Coronary Artery Risk Development in Young Adults (CARDIA) cohort study reported that weight maintenance over time (both normal weight and overweight) in young adults protects against cardiovascular risk, but weight gain increases the risk [5]. Thus, interventions focused on prevention of weight gain in overweight young adults may help prevent obesity and its associated health consequences [6].

Coordinated prevention approaches aimed at improving detrimental lifestyle behaviors have been proposed to prevent obesity [7,8]. Compared with other age groups, young adults eat the least amount of fruits and vegetables [9,10], drink the most sugar-sweetened beverages (SSB) [11], more frequently eat food prepared outside the home (ie, takeout food) [12], and demonstrate declines in physical activity [13-15]. These adverse behavioral lifestyle choices predict excessive weight gain and increased risk of chronic disease later in life [16].

Several recent prevention programs have shown short-term efficacy in young adults to prevent further weight gain [17], but few investigated the use of mHealth (ie, mobile or cellular phone) technology. Advantages of such technology include its wide reach and, once created, its low costs compared with health professional time. The 18- to 29-year-old age group is also the most likely age group to own a mobile phone, with 83% ownership in the US [18]. Interventions delivered via short message service (SMS) text messaging show promise in positively impacting health-related behavior change [19,20]. Our previous pilot study demonstrated the feasibility of delivering an mHealth lifestyle program [21]. Participants in the intervention group decreased their body weight and SSB intake and increased their physical activity and vegetable consumption, although changes were not significant. Qualitative feedback facilitated improvements to the program and informed the development of the TXT2BFiT mHealth program aimed at improving weight management and weight-related dietary and physical activity behaviors among young adults.

Here we report on the efficacy of a randomized controlled trial (RCT) of a larger mHealth lifestyle program, TXT2BFiT, among young adults deemed at high risk for development of obesity. We hypothesized that compared with young adults assigned to a control condition, those who received the TXT2BFiT mHealth intervention would maintain or lose a modest amount of weight and improve lifestyle behaviors.

## Methods

### Overview

The TXT2BFiT study is a two-arm, parallel-design RCT in 18- to 35-year-olds recruited from the Greater Sydney Area, NSW, Australia, between November 2012 and July 2014. All study materials were designed specifically for use in this study only. The trial was approved by the University Human Research Ethics Committee in September 2012 (approval number 15226) and all the participants gave written informed consent. The trial is registered with the Australian New Zealand Clinical Trials Registry (ACTRN12612000924853). Both the protocol and recruitment methods have been previously published [22]. A concise description appears below.

### Subjects

Participants who responded to recruitment materials were directed to complete an online screener survey. Eligible participants were deemed at risk of excess weight gain if they met the following inclusion criteria: had a BMI of 25.0 to 31.9 kg/m^2^, or 23.0 to 24.9 kg/m^2^with reported weight gain greater than 2 kg over the previous 12 months; had a fruit intake of less than two servings daily; had a vegetable intake of less than five servings daily; had an SSB intake of at least 1 L weekly; had energy-dense meals prepared away from home (ie, takeout food) more than once per week; and/or engaged in moderate-intensity physical activity of less than 60 minutes daily. Individuals were excluded if they were pregnant or planning to fall pregnant within the study period, were enrolled in an alternate weight loss program, had lost greater than 10 kg in the past 3 months, taken medications that have caused weight gain of greater than 2 kg, had medical conditions that precluded following dietary or physical activity recommendations, and/or did not speak English. Participants were also required to have a mobile phone capable of receiving text messages and accessing the Internet at least once a week.

Based on our previous meta-analysis [6], it appeared that a difference of 1.7 kg could be expected. The sample size required for detection of a difference of 2 kg with 80% power, significance level of .05, 10 kg standard deviation, and a correlation between baseline and final weight of .8, was 354 subjects after allowing for a 20% dropout rate. Due to a slower-than-expected recruitment rate, and with time and funding constraints, recruitment was stopped at 250 participants.

### Recruitment

Recruitment occurred via letters of invitation from participating general practitioners (GPs) (ie, primary care physicians) in two Medicare Locals—Australian primary health care services units responsible for coordinating care over specified geographic areas—or via electronic or print advertisements, including Facebook and Google (ie, social media and advertising), university electronic newsletters, printed posters, mailbox drops, and newspapers. Participants provided informed written consent. Young adults were compensated for their participation by receiving gift vouchers for completing a 12-week online survey and attending an in-person weigh-in.

### Randomization

A random sequence was generated by an independent researcher and concealed from those responsible for enrolling participants into the intervention arm. Eligible participants were randomized in a 1:1 ratio into intervention and control arms. Randomization was based on a stratified randomized block design, where the strata were the GP clinic and participant gender. While participants were aware of another arm to the trial, every attempt was made to ensure that the nature of this other arm was not revealed.

### Measurements

Demographic characteristics were collected by online survey and included age, gender, postcode (for categorizing socioeconomic status [23]), ethnicity (language spoken at home [24]), education level [24], and income in Australian Dollars (AUD) [24]. Body weight (kg) and height (cm) data were collected to calculate BMI (kg/m^2^) at baseline via both measured and self-report methods. Participants' GPs used a standardized protocol to measure body weight to the nearest 0.1 kg and height to the nearest 0.1 cm at baseline [25]. Participants in both arms were invited for an optional in-person body weight (kg) and height (cm) measurement at the University Metabolic Facility within a 2-week window following the 12-week intervention completion (ie, weeks 13 and 14). Measures were taken by two higher-degree research students blinded to participant allocation.

Online surveys were administered at baseline and within a 2-week window following the 12-week intervention completion (ie, weeks 13 and 14). Data collected included self-reported weight (kg) and height (cm); short categorical questions to assess usual weekly intake of SSB [26], daily intake of fruits and vegetables [26], and weekly takeout meals [27]; and questions about physical activity in the previous 7 days using the short-form International Physical Activity Questionnaire (IPAQ) [28]. The IPAQ was scored using established methods [29] and data were reported as a continuous measure in metabolic equivalent of task (MET)-minutes per week. All data were reported by participants via the online deidentified survey website, SurveyMonkey, from which data were downloaded for analysis.

Engagement with the intervention was assessed using text message replies and number of coaching calls completed. Intervention participants were asked to reply "OK" to 16 messages in the 12 weeks and control participants were asked to reply "OK" to all 4 text messages. Text message delivery reports were created from the text message service provider, My MessageMedia, for delivery status and replies. Detailed records of all coaching calls were collated in a database. The 12-week postintervention survey also asked participants about their access to, and use of, program materials.

### TXT2BFiT Program

**Figure 1:**
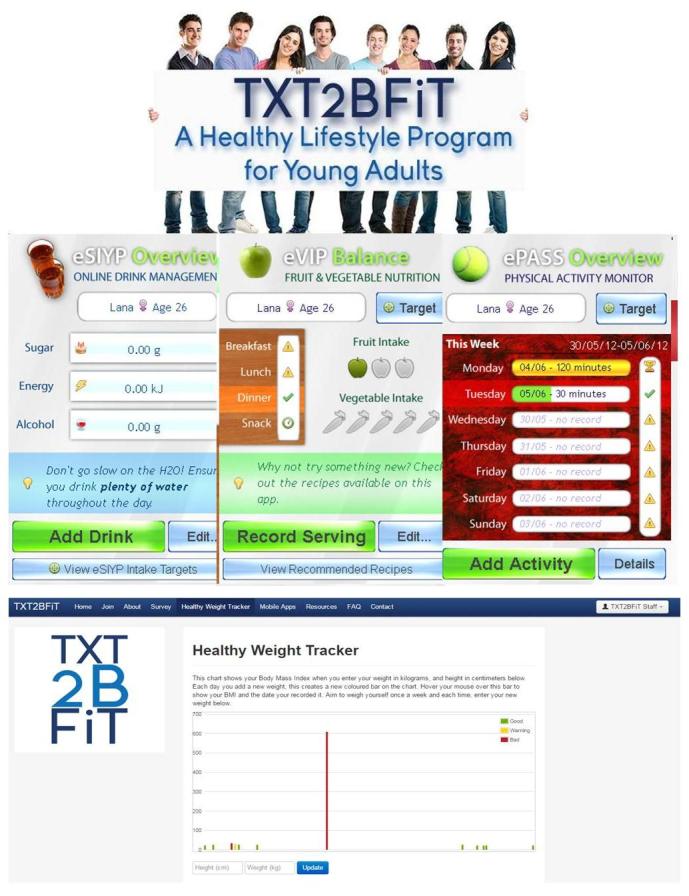
TXT2BFiT program screenshots.

The 12-week intervention program comprised the following: 8 weekly motivational text messages based on the transtheoretical model of behavior change, whereby messages were matched to stage-of-change for each of the individual lifestyle behaviors; 5 personalized coaching calls; weekly emails; and password-protected access to purpose-designed mobile phone apps that provided education and allowed self-monitoring [30], community blog, and support resources available on a password-protected website designed for the study [31] (see Figure 1). Support resources included “easy, healthy eating on a budget,” “emergency meal tool kit,” “meal planning worksheet,” “commit yourself: physical activity planner,” “tips for take-out meals,” “seasonal guide to fruit and vegetables,” and “staying healthy over the holidays.” Text messages were scheduled by two higher-degree research students. The text messages, based on the transtheoretical model of behavior change [32], consisted of 2 per week for each of the four behaviors—SSB, fruits and vegetables, physical activity, and food prepared away from home/takeout—for a total of 8 messages, weekly, tailored to the participant's stage of readiness to change [22] and sent using the My MessageMedia program. Two accredited practicing dietitians conducted the coaching calls according to a standardized protocol and allowed the participants to set goals, and to discuss barriers, enablers, and their progress. Each call lasted approximately 10 to 15 minutes, with 25 minutes allocated for the initial coaching call. The mobile phone apps were educational, for example, providing nutritional information on SSB and takeout meals, providing serving sizes for fruits and vegetables, and allowing self-monitoring of participants' behavior. One email was sent each week reiterating the information in the text messages and included links to the mobile phone apps to remind participants.

Intervention participants were also mailed a printed 18-page booklet containing the two-page control handout summarizing the Australian National Dietary and Physical Activity Guidelines [33,34]. Additional information included sample meal plans, recommendations for daily servings from the core food groups with example serving sizes [34], and information about the four target behaviors addressed by the program—physical activity and sedentary behavior, intake of fruits and vegetables, intake of energy-dense takeout meals prepared away from home, and SSB intake.

Control participants received the mailed two-page handout, the introductory call at week 0 to introduce the program (no coaching given), 4 text messages (one every 3 weeks, during weeks 1 to 12) that restated information in the handout, and access to a website with only electronic versions of the two-page handout, consent form, study information statement, and contact information.

### Statistical Analysis

The primary outcomes, body weight (kg) and BMI (kg/m^2^) at 12 weeks, were compared between the two groups using analysis of covariance models adjusting for baseline values, GP clinic, and gender. Secondary outcomes that were continuous—physical activity MET-minutes and physical activity days—were also analyzed using analysis of covariance models. Robust regression models were used for analyses where residuals indicated nonnormality. Secondary outcomes that were categorical—fruit and vegetable servings per day, SSB consumption per week, and energy-dense takeout meal intake per week—were analyzed using Mantel-Haenszel chi-square tests stratified by GP clinic and gender. The analysis used the "intention-to-treat" principle with multiple imputations to account for missing data. Five imputed datasets were created and the results for continuous outcomes pooled using Rubin’s rules. Chi-square statistics were pooled, and *P* values estimated, using the method described by Li et al [35]. A *P* value <.05 was considered statistically significant. Researchers analyzing participant outcomes were blinded to participant allocation.

We also compared baseline characteristics and baseline primary or secondary outcomes between completers and noncompleters, and between in-person weigh-in attenders and nonattenders, using chi-square tests for categorical variables and independent-sample *t* tests for continuous variables. We compared self-reported weight and BMI with measured values using paired *t* tests. Analyses were performed using SPSS version 22.0 (IBM Corp, Armonk, NY, USA), Stata Statistical Software: Release 13 (Stata Corp, College Station, TX, USA), and SAS version 9.2 (SAS Institute Inc, Cary, NC, USA) on the full intention-to-treat sample.

## Results

### Participant Flow and Attrition

**Figure 2:**
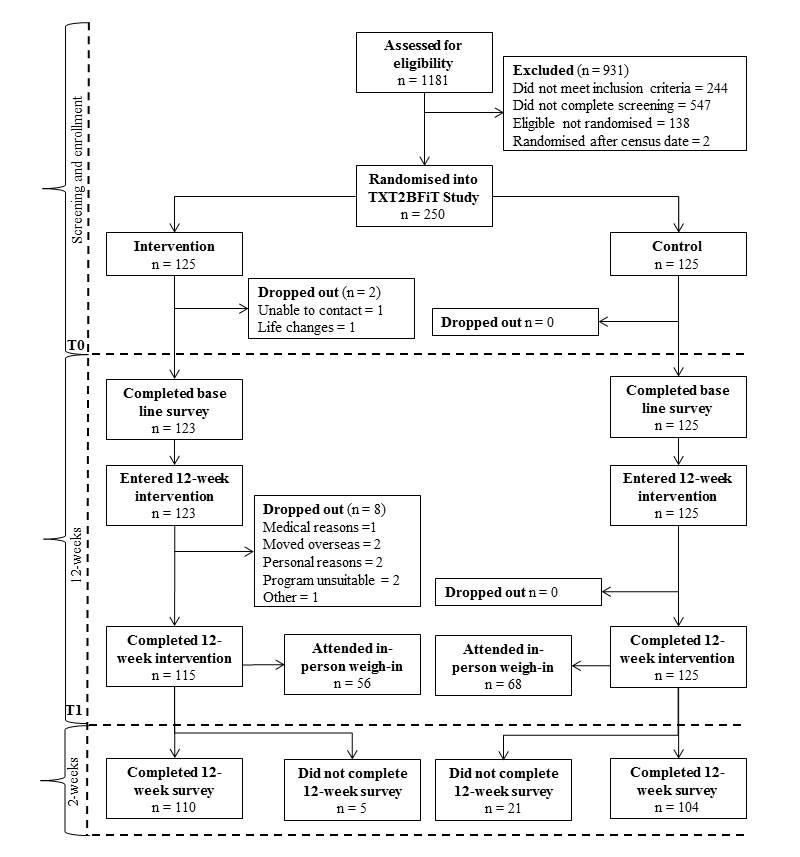
Flow diagram of participants in the TXT2BFiT study from week 0 to week 12.

Recruitment resulted in 1181 enquires, of which 78.83% (931/1181) were excluded or failed to complete screening requirements (see Figure 2). A total of 250 young adults were randomly assigned to the intervention or control group. A total of 10 participants out of 125 (8.0%) dropped out of the intervention group during the 12-week intervention. Reasons for dropping out were as follows: 1 for lack of contact, 1 for life changes, 1 for medical reasons, 2 for personal reasons, 2 moved overseas, 2 found the program unsuitable, and 1 for other reasons not stated. An additional 5 participants out of 125 (4.0%) failed to complete the postintervention online surveys in the intervention group (see Figure 2). No participants dropped out of the control group, and 21 participants out of 125 in the control group (16.8%) did not complete the postintervention online surveys. Completers and noncompleters did not differ significantly in allocation, baseline demographic characteristics, or baseline primary or secondary outcomes (*P*>.11), except for noncompleters who consumed more takeout meals at baseline (*P*=.004). Nearly half of all participants (124/250, 49.6%) accepted the invitation for in-person weight and height measurements—intervention, 56/125, 44.8%; control, 68/125, 54.4%. There were no significant differences in baseline characteristics between participants that attended the in-person weight and height measurements and those that did not (*P*>.34), except that those attending ate less fruit at baseline (*P*=.03).

### Baseline Characteristics

Baseline characteristics of participants are shown in Table 1. Participants in the total randomized sample were mostly older (30 years or older, 107/248, 43.1%), female (152/248, 61.3%), English-speaking only (172/248, 69.4%), highly educated (153/248, 61.7%), and living in a socioeconomically advantaged area (187/248, 75.4%). Participants were overweight on the basis of BMI classification (intervention, 27.3 kg/m^2^; control, 27.1 kg/m^2^) (see Tables 2 and 3). Table 4 shows that, by design, most participants did not meet the recommended servings of fruit (intervention, 82/123, 66.7%; control, 77/125, 61.6%) or servings of vegetables (intervention, 116/123, 94.3%; control, 121/125, 96.8%) [34]; consumed more than 1 L of SSB per week (intervention, 16/123, 13.0%; control, 22/125, 17.6%); and consumed two or more takeout meals per week (intervention, 75/123, 61.0%; control, 79/125, 63.2%). All participants reported above-average levels of recommended physical activity [36] (intervention, 1619.9 MET-minutes per week; control, 1646.8 MET-minutes per week) (see Table 5).

**Table 1:** Baseline demographic characteristics for all randomized participants in the TXT2BFiT study by allocation (n=248)^a^.

Characteristic		Intervention group (n=123)^a^, mean (SD) or n (%)	Control group (n=125), mean (SD) or n (%)
Age in years, mean (SD)		28.1 (4.9)	27.2 (4.9)
**Gender, n (%)**			
	Male	50 (40.7)	46 (36.8)
	Female	73 (59.3)	79 (63.2)
**SES** ^b^ **quintile, n (%)**			
	0-60^c^	8 (6.5)	7 (5.6)
	61-80	28 (22.8)	17 (13.6)
	81-100 (highest)	87 (70.7)	101 (80.8)
**Ethnicity, n (%)**			
	English speaking	82 (66.7)	90 (72.0)
	European	14 (11.4)	11 (8.8)
	Asian	19 (15.4)	19 (15.2)
	Other^d^	8 (6.5)	5 (4.0)
**Education level, n (%)**			
	High school or below	27 (22.0)	21 (16.8)
	Some university or technical school	22 (17.8)	25 (20.0)
	University bachelor degree or higher	74 (60.2)	79 (63.2)
**Weekly income (AUD** ^e^ **), n (%)**			
	Nil or negative	9 (7.3)	13 (10.4)
	$1-499	36 (29.3)	30 (24.0)
	$500-999	19 (15.4)	25 (20.0)
	$1000-1499	36 (29.3)	26 (20.8)
	$1500-1999	14 (11.4)	22 (17.6)
	≥ $2000	9 (7.3)	9 (7.2)

^a^All participants had measured variables including 2 participants who did not complete baseline self-report surveys.

^b^Socioeconomic status (SES).

^c^Bottom-three SES quintiles collapsed.

^d^Pacific Islander and Arabic ethnicities collapsed.

^e^Australian Dollar (AUD).

**Table 2:** Effect of the TXT2BFiT program on measured weight and BMI outcomes for all randomized participants in the study by allocation (intention-to-treat analysis) (n=250).

Measured variable	Intervention group (n=125)^a^, mean (SD)		Control group (n=125), mean (SD)		Model β^b^ (95% CI)	*P*
	Baseline	12 weeks	Baseline	12 weeks		
Body weight, measured in kg	78.3 (11.4)	76.4 (11.1)	79.3 (12.7)	79.5 (12.4)	2.2 (0.8-3.6)	.005
BMI^c^, measured in kg/m^2^	27.3 (2.4)	26.4 (1.9)	27.1 (2.7)	26.8 (2.2)	0.5 (0.1-1.0)	.02

^a^All participants had measured variables including 2 participants who did not complete baseline self-report surveys.

^b^Model coefficients and *P* values were obtained from analysis of covariance models adjusting for baseline values, general practitioner clinic, and gender. Missing baseline and follow-up values were imputed to create five datasets and results were pooled using Rubin’s rules.

^c^Body mass index (BMI).

**Table 3:** Effect of the TXT2BFiT program on self-reported weight and BMI outcomes for all randomized participants in the study by allocation (intention-to-treat analysis) (n=248)^a^.

Self-reported variable	Intervention group (n=123)^a^, mean (SD)		Control group (n=125), mean (SD)		Model β^b^ (95% CI)	*P*
	Baseline	12 weeks	Baseline	12 weeks		
Body weight, self-reported in kg	78.4 (11.2)	76.2 (10.7)	79.3 (12.6)	79.1 (12.8)	2.1 (1.4-2.8)	<.001
BMI^c^, self-reported in kg/m^2^	27.3 (2.3)	26.5 (2.3)	27.0 (2.7)	26.9 (2.5)	0.6 (0.3-1.0)	<.001

^a^All participants had measured variables including 2 participants who did not complete baseline self-report surveys.

^b^Model coefficients and *P* values were obtained from analysis of covariance models adjusting for baseline values, general practitioner clinic, and gender. Missing baseline and follow-up values were imputed to create five datasets and results were pooled using Rubin’s rules.

^c^Body mass index (BMI).

**Table 4:** Effect of the TXT2BFiT program on secondary outcomes for diet for all randomized participants in the study by allocation (intention-to-treat analysis) (n=248)^a^.

Variable^b^		Intervention group (n=123)^a^, n (%)		Control group (n=125), n (%)		*P*
		Baseline	12 weeks	Baseline	12 weeks	
**Fruit servings** ^c^ **per day**						
	≤1	82 (66.7)	30 (24.4)	77 (61.6)	50 (40.0)	.18
	2	31 (25.2)	75 (61.0)	31 (24.8)	55 (44.0)	
	≥3	10 (8.1)	18 (14.6)	17 (13.6)	20 (16.0)	
**Vegetable servings** ^d^ **per day**						
	≤1	35 (28.5)	12 (9.8)	34 (27.2)	25 (20.0)	.009
	2	46 (37.4)	32 (26.0)	46 (36.8)	40 (32.0)	
	3	23 (18.7)	36 (29.3)	27 (21.6)	32 (25.6)	
	≥4	19 (15.4)	43 (35.0)	18 (14.4)	28 (22.4)	
**SSB** ^e^ **intake per week in mL**						
	Nil	22 (17.9)	37 (30.1)	33 (26.4)	32 (25.6)	.002
	ASD^f^	27 (22.0)	32 (26.0)	17 (13.6)	15 (12.0)	
	<500	37 (30.1)	45 (36.6)	31 (24.8)	43 (34.4)	
	500-999	21 (17.1)	8 (6.5)	22 (17.6)	26 (20.8)	
	≥1000	16 (13.0)	1 (0.8)	22 (17.6)	9 (7.2)	
**Takeout meal intake per week**						
	Nil	3 (2.4)	3 (2.4)	2 (1.6)	8 (6.4)	.01
	≤1	45 (36.6)	85 (69.1)	44 (35.2)	60 (48.0)	
	2-3	58 (47.2)	28 (22.8)	53 (42.4)	37 (29.6)	
	4-5	11 (8.9)	5 (4.1)	21 (16.8)	17 (13.6)	
	6-7	6 (4.9)	2 (1.6)	5 (4.0)	3 (2.4)	

^a^All participants had measured variables including 2 participants who did not complete baseline self-report surveys.

^b^All questions were asked for average daily or weekly intake over the previous month. *P* values were adjusted for practice and gender. All variables were analyzed using Mantel-Haenszel chi-square tests stratified by general practitioner clinic and gender. Five imputed datasets were created and the results for the chi-square statistics were pooled, and *P* values estimated, using the method described by Li et al [35].

^c^One serving of fruit is equivalent to one medium piece (eg, one apple or one orange), two small pieces (eg, two plums), or one cup of diced pieces (fresh or canned).

^d^One serving of vegetables is equivalent to half a cup of cooked vegetables (fresh, frozen, or canned) or one cup of raw salad vegetables.

^e^Sugar-sweetened beverages (SSB).

^f^Artificially sweetened drinks (ASD).

**Table 5:** Effect of the TXT2BFiT program on secondary physical activity outcomes from the IPAQ^a^for all randomized young adults in the study by allocation (intention-to-treat analysis) (n=248)^b^.

Variable		Intervention group (n=123)^b^, mean (SD)		Control group (n=125), mean (SD)		Model β^c^(95% CI)	*P*
		Baseline	12 weeks	Baseline	12 weeks		
**Vigorous physical activity**							
	MET^d^-minutes per week	758.6 (1112.0)	1006.1 (1463.6)	840.0 (1072.1)	944.1 (958.3)	-20.0 (-195.9 to 155.9)	.80
	Days per week	1.5 (1.6)	2.1 (1.7)	1.8 (1.8)	2.0 (1.7)	-0.3 (-0.7 to 0.2)	.20
**Walking physical activity**							
	MET-minutes per week	691.9 (867.5)	927.3 (1163.0)	630.0 (595.3)	777.3 (828.7)	-69.8 (-180.2 to 40.6)	.20
	Days per week	4.3 (2.0)	5.2 (1.9)	4.6 (2.2)	4.7 (2.2)	-0.6 (-1.1 to -0.1)	.02
**Moderate physical activity**							
	MET-minutes per week	169.4 (359.8)	258.7 (417.9)	176.8 (393.9)	170.8 (222.4)	8.0 (-34.3 to 50.5)	.70
	Days per week	0.8 (1.2)	1.4 (1.6)	0.9 (1.3)	1.0 (1.3)	-0.4 (-0.7 to 0.1)	.10
**Total physical activity**							
	MET-minutes per week	1619.9 (1581.1)	2192.4 (2133.1)	1646.8 (1474.6)	1892.7 (1539.3)	-252.5 (-503.8 to -1.2)	.05
	Days per week	6.6 (3.3)	8.8 (3.6)	7.4 (3.8)	7.7 (3.6)	-1.3 (-2.2 to -0.5)	.003

^a^International Physical Activity Questionnaire (IPAQ).

^b^All participants had measured variables including 2 participants who did not complete baseline self-report surveys.

^c^Model coefficients and *P* values were obtained from analysis of covariance models adjusting for baseline values, general practitioner clinic, and gender. Robust regression models were used for analyses where residuals indicated nonnormality. Missing baseline and follow-up values were imputed to create five datasets and results were pooled using Rubin’s rules.

^d^Metabolic equivalent of task (MET).

### Engagement With the Program

The mean number of coaching calls completed in the intervention group was 4.6 (SD 1.1) out of 5 (82.4% overall completed all 5). All participants who completed the postintervention survey reported engaging with coaching calls. Of the 12,308 text messages sent during the 12-week intervention (control, 500; intervention 11,808), only 2.27% (280) were not delivered (control, 15/500, 3.0%; intervention, 265/11,808, 2.24%). Over half (66/123, 53.7%) of the intervention participants replied to 8 or more of the 16 SMS text messages with a requested response, with 25 of the 123 participants (20.3%) replying to all. Most control participants replied to 2 or more of the 4 text messages (114/125, 91.2%), with 62.4% (78/125) replying to all 4 of them. A total of 100 of the 110 (90.9%) intervention participants who completed the follow-up survey self-reported that they used the SMS text messages. Email delivery was 100%, with 84 of 110 (76.4%) participants reporting that they used the email messages during the study. A total of 82 out of 110 (74.5%) intervention participants reported that they did not access the mobile phone apps during the study. The mailed booklet was used by 72 of the 110 (65.5%) intervention participants and only 7 out of 110 (6.4%) used the blog. Most intervention participants (65/110, 59.1%) did not use the resources available on the website. Of those that did, the takeout meal planner was reported as most used by the intervention participants (28/110, 25.5%).

### Body Weight (kg) and BMI (kg/m^2^)

Young adults in the intervention group were 2.2 kg lighter at 12 weeks compared to the control group using measured body weight after adjusting for baseline-measured body weight (95% CI 0.8-3.6, *P*=.005) (see Table 2). A similar pattern was observed with BMI, which was 0.5 kg/m^2^less at 12 weeks (95% CI 0.1-1.0, *P*=.02) for the intervention group compared to the control group using measured BMI.

Using self-reported body weight measures, intervention participants were 2.1 kg (95% CI 1.4-2.8, *P*<.001) and 0.6 BMI units (kg/m^2^) (95% CI 0.3-1.0, *P*<.001) lighter than control participants at 12 weeks (see Table 3).

At baseline, there was no significant difference between measured and self-reported weight and BMI (248/250, 99.2%) (*P*>.11). At 12 weeks, among participants with a measured weight (124/250, 49.6%), average self-reported weight was 0.7 kg (SD 1.3) less than the measured weight (*P*<.001). However, there was no difference between intervention (56/125, 44.8%; 0.8 kg, SD 1.2) and control groups (68/125, 54.4%; 0.6 kg, SD 1.4) (*P*=.44). There was no difference between measured and self-reported BMI at 12 weeks (*P*=.26).

### Fruit and Vegetable Intake

The majority of participants reported consuming the recommended two servings of fruit per day or more after 12 weeks (see Table 4), with a nonsignificant difference between intervention group and control group (*P*=.18). Intervention participants were more likely to consume greater quantities of vegetables after 12 weeks compared to control participants (*P*=.009). For example, 35.0% (43/123) of intervention participants consumed four or more servings of vegetables compared to 22.4% (28/125) of control participants.

### Sugar-Sweetened Beverage and Takeout Meal Intake

Intervention participants consumed SSB less frequently after 12 weeks compared with the control participants (*P*=.002) (see Table 4). For example, 92.7% (114/123) of intervention participants consumed 500 mL or less of SSB compared to 72.0% (90/125) of control participants at 12 weeks.

After 12 weeks, intervention participants reported consuming energy-dense takeout meals less frequently during the week compared with the control participants—54.4% (68/125) of intervention participants compared to 71.5% (88/123) of control participants consumed one or fewer energy-dense takeout meals per week (*P*=.01) (see Table 4).

### Physical Activity

Intervention participants reported a mean increase of 563.1 (SD 1983.6) MET-minutes per week after 12 weeks. Control participants reported a mean increase of 244.4 (SD 1510.6) MET-minutes per week (see Table 5). These observed increases in energy expenditure were predominantly due to increased reported vigorous and walking activities, which increased by an average 243.0 (SD 1073.3) and 231.8 (SD 1313.9) MET-minutes per week among intervention participants, respectively, and 102.5 (SD 1148.6) and 148.1 (SD 747.3) MET-minutes per week among control participants, respectively. After adjusting for baseline MET-minutes per week, GP clinic, and gender there was a significant effect of the intervention on average MET-minutes per week at 12 weeks (95% CI -503.8 to -1.2, *P*=.05). Total and walking physical activity days increased more in the intervention group (95% CI -2.2 to -0.5, *P*=.003) compared to the control group (95% CI -1.1 to -0.1, *P*=.02).

## Discussion

### Principal Findings

This 12-week TXT2BFiT mHealth intervention was effective in preventing unhealthy weight gain, resulting in modest weight loss and improvement in lifestyle behaviors. Compared with control participants, intervention participants consumed more vegetables and less SSB, consumed fewer energy-dense meals prepared away from home, and increased their physical activity, with increased total and walking days of physical activity. As far as we are aware, this is the first reported trial of a multi-component mobile phone-based program conducted in young adults.

Participants in the intervention program weighed 2.2 kg less than control participants at 12 weeks. The prevention of weight gain is an important public health priority for this population, given the likelihood of weight gain reported by prior observational studies in young adult populations [37,38]. Furthermore, young adults have been born into an increasingly "obesogenic environment" and are at a greater risk of becoming obese [4]. If there is no effort to change these behavioral patterns, it is likely that young adults and subsequent generations will have a higher incidence of overweight and obesity.

A greater number of intervention participants reported increasing vegetable servings compared with controls. While recommendations for increased fruits and vegetables alone may not prevent weight gain [39], intervention participants also reported reductions in energy-dense meals prepared away from home and in SSB intake, and further increased their physical activity.

Most of the recent weight gain prevention interventions in young adults have targeted improvements in healthy eating and physical activity through in-person group interventions [17]. Two previous intervention studies in young adults were conducted via an online tutorial-style platform [40,41]. Green et al conducted a 3-month online curriculum-designed program based on nondiet principles, with weekly goal setting to increase fruit and vegetable intake and increase physical activity [40]. Intervention participants increased fruit and vegetable intake and increased physical activity compared to controls, however, no significant change in weight outcomes resulted. A shorter social cognitive theory-based intervention of 6 weeks by Gow et al focused on diet and physical activity habits pertinent to the transition period to college [41]. BMI was lower in the intervention group participating in the online activities and receiving weekly emails, with no effects on diet and physical activity outcomes. Other studies investigating the use of technology in the prevention of weight gain for young adults have published protocol papers, but there have been no reports of efficacy to date [42].

This study was innovative in the use of text messages, already demonstrated as successful in older adults in combination with coaching telephone calls [43]. This study design did not test the efficacy of the individual components, but the engagement data suggested that the coaching calls and text messages were useful to participants, with 100% and 90.9% reporting having used these components, respectively. A text message intervention in normal-weight young adults showed messages based on a habit framework can improve fruit consumption, and simply reminding young adults to be conscious of their food choices may be sufficient to improve their overall vegetable consumption [44]. Costs of delivering a mobile program could be reduced without coaching calls, but our previous pilot intervention did not detect effectiveness in dietary change with text messages alone, without coaching calls.

An important strength of this study was low attrition (14.4% at 12 weeks), and the interventions were delivered according to protocol, with 92% of coaching calls completed and only 2.3% of SMS text messages failing to send. Primary health care facilities and public advertisements increased reach in recruitment. This study also recruited more males than is often expected in studies of this type [45]. Another strength included analyzed outcomes being blinded to treatment allocation and using intention-to-treat principles. The use of GPs to measure height and weight on their scales could introduce measurement error. However, GP clinic was one of the strata for participant randomization, and observer and equipment error would have been distributed equally across groups. Therefore, measurement bias should not have impacted the results from the analysis of covariance. It is acknowledged that using GP scales for baseline weight and clinic scales with a trained dietitian for follow-up measures was not ideal. As self-reported measures were used for all studied outcomes, the data may be biased. Self-report may underestimate weight, but has been shown to accurately identify overweight and/or obesity in the majority of a sample of young people [46]. An element of social desirability might influence reporting of lifestyle behaviors. Both groups were provided dietary and physical activity guidelines, however, greater significant improvements in intervention participants were seen in this study. Further, the sample was mostly well educated and from higher socioeconomic areas, which may influence the generalizability of the results [47].

### Conclusions

In conclusion, intervening in the lives of young adults with unhealthy lifestyle behaviors, who have an increased risk of weight gain and developing obesity, appears to have a beneficial impact on preventing weight gain. While the short-term efficacy of the 12-week TXT2BFiT intervention program is promising, maintenance of outcomes in the longer term will be evaluated at 9 months. The potentially wide reach and low delivery costs of using mHealth, coupled with the growing problem of obesity in younger adulthood, means translation and implementation of this program to the community at large also warrants further consideration.

## Appendix

Multimedia Appendix 1 CONSORT-EHEALTH checklist V1.6.1 [48].

## Abbreviations

ASD: artificially sweetened drinks
AUD: Australian Dollar
BMI: body mass index
CARDIA: Coronary Artery Risk Development in Young Adults
GP: general practitioner
HCF: Hospitals Contribution Fund
IPAQ: International Physical Activity Questionnaire
MET: metabolic equivalent of task
NHMRC: National Health and Medical Research Council
RCT: randomized controlled trial
SES: socioeconomic status
SMS: short messages service
SSB: sugar-sweetened beverages
