# Supplementary material for: Effectiveness of a mHealth Lifestyle Program With Telephone Support (TXT2BFiT) to Prevent Unhealthy Weight Gain in Young Adults: Randomized Controlled Trial
Source: JMIR Mhealth Uhealth. 2015 Jun 15;3(2):e66. doi: 10.2196/mhealth.4530 (PMC4526939; doi:10.2196/mhealth.4530)
Supplement: Multimedia Appendix 1 [file mhealth_v3i2e66_app1.pdf]

CONSORT-EHEALTH (V 1.6.1) - Submission/Publication Form

The CONSORT-EHEALTH checklist is intended for authors of randomized trials evaluating web-based and Internet-based applications/interventions, including mobile interventions, electronic games (incl multiplayer games), social media, certain telehealth applications, and other interactive and/or networked electronic applications. Some of the items (e.g. all subitems under item 5 - description of the intervention) may also be applicable for other study designs.

The goal of the CONSORT EHEALTH checklist and guideline is to be
a) a guide for reporting for authors of RCTs,
b) to form a basis for appraisal of an ehealth trial (in terms of validity)

CONSORT-EHEALTH items/subitems are MANDATORY reporting items for studies published in the Journal of Medical Internet Research and other journals / scientific societies endorsing the checklist.

Items numbered 1., 2., 3., 4a., 4b etc are original CONSORT or CONSORT-NPT (non-pharmacologic treatment) items.
Items with Roman numerals (i., ii, iii, iv etc.) are CONSORT-EHEALTH extensions/clarifications.

As the CONSORT-EHEALTH checklist is still considered in a formative stage, we would ask that you also RATE ON A SCALE OF 1-5 how important/useful you feel each item is FOR THE PURPOSE OF THE CHECKLIST and reporting guideline (optional).

Mandatory reporting items are marked with a red *.
In the textboxes, either copy & paste the relevant sections from your manuscript into this form - please include any quotes from your manuscript in QUOTATION MARKS,
or answer directly by providing additional information not in the manuscript, or elaborating on why the item was not relevant for this study.

YOUR ANSWERS WILL BE PUBLISHED AS A SUPPLEMENTARY FILE TO YOUR PUBLICATION IN JMIR AND ARE CONSIDERED PART OF YOUR PUBLICATION (IF ACCEPTED).
Please fill in these questions diligently. Information will not be copyedited, so please use proper spelling and grammar, use correct capitalization, and avoid abbreviations.

DO NOT FORGET TO SAVE AS PDF _AND_ CLICK THE SUBMIT BUTTON SO YOUR ANSWERS ARE IN OUR DATABASE !!!

Citation Suggestion (if you append the pdf as Appendix we suggest to cite this paper in the caption):
Eysenbach G, CONSORT-EHEALTH Group
CONSORT-EHEALTH: Improving and Standardizing Evaluation Reports of Web-based and Mobile Health Interventions
J Med Internet Res 2011;13(4):e126
URL: http://www.jmir.org/2011/4/e126/
doi: 10.2196/jmir.1923
PMID: 22209829

* Required

Your name *

First Last

Stephanie Partridge

Primary Affiliation (short), City, Country *

University of Toronto, Toronto, Canada

The University of Sydney

Your e-mail address *

abc@gmail.com

stephanie.partridge@sydney.

Title of your manuscript *

Provide the (draft) title of your manuscript.

"m-Health lifestyle program with telephone support (TXT2BFiT) prevents unhealthy weight gain in young adults: 12-week efficacy outcomes of a randomised controlled trial"

Article Preparation Status/Stage *

At which stage in your article preparation are you currently (at the time you fill in this form)

- ○ not submitted yet - in early draft status
- ◉ not submitted yet - in late draft status, just before submission
- ○ submitted to a journal but not reviewed yet
- ○ submitted to a journal and after receiving initial reviewer comments
- ○ submitted to a journal and accepted, but not published yet
- ○ published
- ○ Other:

Journal *

If you already know where you will submit this paper (or if it is already submitted), please provide the journal name (if it is not JMIR, provide the journal name under "other")

- ○ not submitted yet / unclear where I will submit this
- ◉ Journal of Medical Internet Research (JMIR)
- ○ Other:

Manuscript tracking number *

If this is a JMIR submission, please provide the manuscript tracking number under "other" (The ms

tracking number can be found in the submission acknowledgement email, or when you login as author in JMIR. If the paper is already published in JMIR, then the ms tracking number is the four-digit number at the end of the DOI, to be found at the bottom of each published article in JMIR)

- (selected) no ms number (yet) / not (yet) submitted to / published in JMIR
- Other:

TITLE AND ABSTRACT

1a) TITLE: Identification as a randomized trial in the title

1a) Does your paper address CONSORT item 1a? *

I.e does the title contain the phrase "Randomized Controlled Trial"? (if not, explain the reason under "other")

- (selected) yes
- Other:

1a-i) Identify the mode of delivery in the title

Identify the mode of delivery. Preferably use “web-based” and/or “mobile” and/or “electronic game” in the title. Avoid ambiguous terms like “online”, “virtual”, “interactive”. Use “Internet-based” only if Intervention includes non-web-based Internet components (e.g. email), use “computer-based” or “electronic” only if offline products are used. Use “virtual” only in the context of “virtual reality” (3-D worlds). Use “online” only in the context of “online support groups”. Complement or substitute product names with broader terms for the class of products (such as “mobile” or “smart phone” instead of “iphone”), especially if the application runs on different platforms.

	1	2	3	4	5	
subitem not at all important	○	○	○	○	●	essential

Does your paper address subitem 1a-i? *

Copy and paste relevant sections from manuscript title (include quotes in quotation marks "like this" to indicate direct quotes from your manuscript), or elaborate on this item by providing additional information not in the ms, or briefly explain why the item is not applicable/relevant for your study

In title: "m-Health lifestyle program"

1a-ii) Non-web-based components or important co-interventions in title

Mention non-web-based components or important co-interventions in title, if any (e.g., “with telephone support”).

	1	2	3	4	5	
subitem not at all important	○	○	○	○	◉	essential

Does your paper address subitem 1a-ii?

Copy and paste relevant sections from manuscript title (include quotes in quotation marks "like this" to indicate direct quotes from your manuscript), or elaborate on this item by providing additional information not in the ms, or briefly explain why the item is not applicable/relevant for your study

In title: "with telephone support"

1a-iii) Primary condition or target group in the title

Mention primary condition or target group in the title, if any (e.g., "for children with Type I Diabetes") Example: A Web-based and Mobile Intervention with Telephone Support for Children with Type I Diabetes: Randomized Controlled Trial

	1	2	3	4	5	
subitem not at all important	○	○	○	○	◉	essential

Does your paper address subitem 1a-iii? *

Copy and paste relevant sections from manuscript title (include quotes in quotation marks "like this" to indicate direct quotes from your manuscript), or elaborate on this item by providing additional information not in the ms, or briefly explain why the item is not applicable/relevant for your study

In title: "unhealthy weight gain in young adults"

1b) ABSTRACT: Structured summary of trial design, methods, results, and conclusions

NPT extension: Description of experimental treatment, comparator, care providers, centers, and blinding status.

1b-i) Key features/functionalities/components of the intervention and comparator in the METHODS section of the ABSTRACT

Mention key features/functionalities/components of the intervention and comparator in the abstract. If possible, also mention theories and principles used for designing the site. Keep in mind the needs of

systematic reviewers and indexers by including important synonyms. (Note: Only report in the abstract what the main paper is reporting. If this information is missing from the main body of text, consider adding it)

1 2 3 4 5

subitem not at all important ○ ○ ○ ○ ◉ essential

Does your paper address subitem 1b-i? *

Copy and paste relevant sections from the manuscript abstract (include quotes in quotation marks "like this" to indicate direct quotes from your manuscript), or elaborate on this item by providing additional information not in the ms, or briefly explain why the item is not applicable/relevant for your study

In abstract methods section: "In the 12week trial, the intervention (I) received eight text messages weekly based on the Transtheoretical Model of behaviour change, one email weekly, five personalized coaching calls, diet booklet and access to resources and Smartphone applications on a website. Controls (C) received only four text messages and printed dietary and physical activity guidelines."

1b-ii) Level of human involvement in the METHODS section of the ABSTRACT

Clarify the level of human involvement in the abstract, e.g., use phrases like “fully automated” vs. “therapist/nurse/care provider/physician-assisted” (mention number and expertise of providers involved, if any). (Note: Only report in the abstract what the main paper is reporting. If this information is missing from the main body of text, consider adding it)

1 2 3 4 5

subitem not at all important ○ ○ ○ ◉ ○ essential

Does your paper address subitem 1b-ii?

Copy and paste relevant sections from the manuscript abstract (include quotes in quotation marks "like this" to indicate direct quotes from your manuscript), or elaborate on this item by providing additional information not in the ms, or briefly explain why the item is not applicable/relevant for your study

In abstract methods section: "five personalized coaching calls"

1b-iii) Open vs. closed, web-based (self-assessment) vs. face-to-face assessments in the METHODS section of the ABSTRACT

Mention how participants were recruited (online vs. offline), e.g., from an open access website or from a clinic or a closed online user group (closed usergroup trial), and clarify if this was a purely web-based trial, or there were face-to-face components (as part of the intervention or for assessment). Clearly say if outcomes were self-assessed through questionnaires (as common in web-based trials). Note: In

traditional offline trials, an open trial (open-label trial) is a type of clinical trial in which both the researchers and participants know which treatment is being administered. To avoid confusion, use “blinded” or “unblinded” to indicated the level of blinding instead of “open”, as “open” in web-based trials usually refers to “open access” (i.e. participants can self-enrol). (Note: Only report in the abstract what the main paper is reporting. If this information is missing from the main body of text, consider adding it)

	1	2	3	4	5	
subitem not at all important	○	○	○	●	○	essential

Does your paper address subitem 1b-iii?

Copy and paste relevant sections from the manuscript abstract (include quotes in quotation marks "like this" to indicate direct quotes from your manuscript), or elaborate on this item by providing additional information not in the ms, or briefly explain why the item is not applicable/relevant for your study

In abstract methods section: "Measured body weight and height were collected at baseline and 12-weeks. Outcomes were assessed via online surveys at baseline and 12-weeks including self-reported weight, dietary and physical activity measures."

1b-iv) RESULTS section in abstract must contain use data

Report number of participants enrolled/assessed in each group, the use/uptake of the intervention (e.g., attrition/adherence metrics, use over time, number of logins etc.), in addition to primary/secondary outcomes. (Note: Only report in the abstract what the main paper is reporting. If this information is missing from the main body of text, consider adding it)

	1	2	3	4	5	
subitem not at all important	○	○	○	○	●	essential

Does your paper address subitem 1b-iv?

Copy and paste relevant sections from the manuscript abstract (include quotes in quotation marks "like this" to indicate direct quotes from your manuscript), or elaborate on this item by providing additional information not in the ms, or briefly explain why the item is not applicable/relevant for your study

In abstract results section: "Two hundred fourteen (110 I; 104 C) completed the 12-week trial. Ten participants (8.0%; 10 I; 0 C) dropped out and 26 (10.4%; 5 I; 21 C) participants did not complete post intervention online surveys. Adherence to coaching calls and delivery of text messages was over 95%. At 12-weeks the intervention group were 2.2 kg (95% CI 0.8 to 3.6) lighter than controls (P=0.005) and consumed more vegetables (P=0.009), less sugary soft drinks (P = 0.002) and less energy-dense take-out meals (P=0.0014) and increased total physical activity (P=0.05) and total physical activity days (P=0.003)."

1b-v) CONCLUSIONS/DISCUSSION in abstract for negative trials

Conclusions/Discussions in abstract for negative trials: Discuss the primary outcome - if the trial is

negative (primary outcome not changed), and the intervention was not used, discuss whether negative results are attributable to lack of uptake and discuss reasons. (Note: Only report in the abstract what the main paper is reporting. If this information is missing from the main body of text, consider adding it)

	1	2	3	4	5	
subitem not at all important	○	○	○	○	◉	essential

Does your paper address subitem 1b-v?

Copy and paste relevant sections from the manuscript abstract (include quotes in quotation marks "like this" to indicate direct quotes from your manuscript), or elaborate on this item by providing additional information not in the ms, or briefly explain why the item is not applicable/relevant for your study

In abstract conclusion section: "The TXT2BFiT low-intensity intervention was successful in preventing weight gain with modest weight-loss and improvement in lifestyle behaviours among overweight young adults. The short-term success shows potential for translation into a community-based program."

INTRODUCTION

2a) In INTRODUCTION: Scientific background and explanation of rationale

2a-i) Problem and the type of system/solution

Describe the problem and the type of system/solution that is object of the study: intended as stand-alone intervention vs. incorporated in broader health care program? Intended for a particular patient population? Goals of the intervention, e.g., being more cost-effective to other interventions, replace or complement other solutions? (Note: Details about the intervention are provided in “Methods” under 5)

	1	2	3	4	5	
subitem not at all important	○	○	○	○	◉	essential

Does your paper address subitem 2a-i? *

Copy and paste relevant sections from the manuscript (include quotes in quotation marks "like this" to indicate direct quotes from your manuscript), or elaborate on this item by providing additional information not in the ms, or briefly explain why the item is not applicable/relevant for your study

In introduction section: "More than 35% of adults globally are overweight or obese and in developed countries, the peak prevalence of obesity is moving to younger ages [1]. For example, younger Americans and Australians are gaining more weight than any other adult age group [2-4]. As body mass index (BMI) exceeds 23 kg m-2, risks of cardiovascular disease, certain cancers, diabetes, osteoarthritis, and chronic kidney disease increases [1]. The CARDIA cohort study reported weight maintenance over time (both normal weight and overweight) in young adults protects against cardiovascular risk but weight gain increases the risk [5]. Thus, interventions focused on prevention of weight gain in overweight young adults may help prevent obesity and its associated health consequences [6]."

2a-ii) Scientific background, rationale: What is known about the (type of) system

Scientific background, rationale: What is known about the (type of) system that is the object of the study (be sure to discuss the use of similar systems for other conditions/diagnoses, if appropiate), motivation for the study, i.e. what are the reasons for and what is the context for this specific study, from which stakeholder viewpoint is the study performed, potential impact of findings [2]. Briefly justify the choice of the comparator.

	1	2	3	4	5	
subitem not at all important	○	○	○	○	◉	essential

Does your paper address subitem 2a-ii? *

Copy and paste relevant sections from the manuscript (include quotes in quotation marks "like this" to indicate direct quotes from your manuscript), or elaborate on this item by providing additional information not in the ms, or briefly explain why the item is not applicable/relevant for your study

In introduction section: "Co-ordinated prevention approaches aimed at improving detrimental lifestyle behaviours have been proposed to prevent obesity [7, 8]. Compared with other age groups, young adults eat the least fruit and vegetables [9, 10], drink the most sugary sweetened beverages (SSB) [11], more frequently eat food prepared outside the home (take-out) [12] and demonstrate declines in physical activity [13-15]. These adverse behavioural lifestyle choices predict excessive weight gain and increased risk of chronic disease later in life [16].
Several recent prevention programs have shown short-term efficacy in young adults to prevent further weight gain [17], but few investigated the use of m-Health (mobile or cellular phone) technology. Advantages of such technology include its wide-reach, and once created, low costs compared with health professional time. The 18 to 29 year-old age group are also the most likely age group to own a Smartphone, with 83% ownership in the US [18] and interventions delivered via short message service (SMS) text messaging show promise in positively impacting health-related behaviour change [19, 20]. Our previous pilot study demonstrated the feasibility of delivering an m-Health lifestyle program [21]. Participants in the intervention group decreased their body weight and SSB intake and increased their physical activity and vegetable consumption, although changes were not significant. Qualitative feedback facilitated improvements to the program and informed the development of the TXT2BFiT m-Health program aimed at improving weight management and weight-related dietary and physical activity behaviours among young adults."

2b) In INTRODUCTION: Specific objectives or hypotheses

Does your paper address CONSORT subitem 2b? *

Copy and paste relevant sections from the manuscript (include quotes in quotation marks "like this" to indicate direct quotes from your manuscript), or elaborate on this item by providing additional information not in the ms, or briefly explain why the item is not applicable/relevant for your study

In introduction section: "Here we report on the efficacy of a randomised controlled trial (RCT) of a larger m-Health lifestyle program, 'TXT2BFiT' among young adults deemed at high risk for development of obesity. We hypothesized that compared with young adults assigned to a control condition, those who received the TXT2BFiT m-Health intervention would maintain or lose a modest amount of weight and improve lifestyle behaviours."

METHODS

3a) Description of trial design (such as parallel, factorial) including allocation ratio

Does your paper address CONSORT subitem 3a? *

Copy and paste relevant sections from the manuscript (include quotes in quotation marks "like this" to indicate direct quotes from your manuscript), or elaborate on this item by providing additional information not in the ms, or briefly explain why the item is not applicable/relevant for your study

In methods section: "The TXT2BFiT study is a two arm parallel design RCT in 18 to 35 year olds recruited from the Greater Sydney Area NSW, Australia."

3b) Important changes to methods after trial commencement (such as eligibility criteria), with reasons

Does your paper address CONSORT subitem 3b? *

Copy and paste relevant sections from the manuscript (include quotes in quotation marks "like this" to indicate direct quotes from your manuscript), or elaborate on this item by providing additional information not in the ms, or briefly explain why the item is not applicable/relevant for your study

In methods section: "Due to slower than expected recruitment rate and with time and funding constraints, recruitment was stopped at 250 participants."

3b-i) Bug fixes, Downtimes, Content Changes

Bug fixes, Downtimes, Content Changes: ehealth systems are often dynamic systems. A description of changes to methods therefore also includes important changes made on the intervention or comparator during the trial (e.g., major bug fixes or changes in the functionality or content) (5-iii) and other “unexpected events” that may have influenced study design such as staff changes, system failures/downtimes, etc. [2].

	1	2	3	4	5	
subitem not at all important	○	○	○	○	●	essential

Does your paper address subitem 3b-i?

Copy and paste relevant sections from the manuscript (include quotes in quotation marks "like this" to indicate direct quotes from your manuscript), or elaborate on this item by providing additional information not in the ms, or briefly explain why the item is not applicable/relevant for your study

Not applicable. No Bug fixes, Downtimes, Content Changes.

4a) Eligibility criteria for participants

Does your paper address CONSORT subitem 4a? *

Copy and paste relevant sections from the manuscript (include quotes in quotation marks "like this" to indicate direct quotes from your manuscript), or elaborate on this item by providing additional information not in the ms, or briefly explain why the item is not applicable/relevant for your study

In methods section: "Participants who responded to recruitment materials were directed to complete an online screener survey. Eligible participants were deemed at risk of excess weight gain if they had a BMI 25.0 to 31.9 kg m-2 , or 23.0 to 24.9 kg m-2 with reported weight gain greater than two kilograms (kg) over previous 12 months; and fruit intake < two serves daily; and/or vegetable intake < five serves daily; and/or SSB intake ≥ one litre (L) weekly; and/or energy-dense meals prepared away from home (take-out) > one weekly; and/or moderate intensity physical activity < 60 minutes daily. Individuals were excluded if they were pregnant or planning to fall pregnant within the study period; enrolled in an alternate weight loss program; had lost greater than 10 kg in the past three months; taken medications that have caused weight gain of greater than two kg; medical conditions that preclude following dietary or physical activity recommendations; and/or did not speak English. Participants were also required to have a mobile phone capable of receiving text messages and access to the internet at least once a week."

4a-i) Computer / Internet literacy

Computer / Internet literacy is often an implicit “de facto” eligibility criterion - this should be explicitly clarified.

	1	2	3	4	5	
subitem not at all important	○	○	○	○	◉	essential

Does your paper address subitem 4a-i?

Copy and paste relevant sections from the manuscript (include quotes in quotation marks "like this" to indicate direct quotes from your manuscript), or elaborate on this item by providing additional information not in the ms, or briefly explain why the item is not applicable/relevant for your study

In methods section: "Participants were also required to have a mobile phone capable of receiving text messages and access to the internet at least once a week."

4a-ii) Open vs. closed, web-based vs. face-to-face assessments:

Open vs. closed, web-based vs. face-to-face assessments: Mention how participants were recruited (online vs. offline), e.g., from an open access website or from a clinic, and clarify if this was a purely web-based trial, or there were face-to-face components (as part of the intervention or for assessment), i.e., to what degree got the study team to know the participant. In online-only trials, clarify if participants were quasi-anonymous and whether having multiple identities was possible or whether technical or logistical measures (e.g., cookies, email confirmation, phone calls) were used to detect/prevent these.

	1	2	3	4	5	
subitem not at all important	○	○	○	○	◉	essential

Does your paper address subitem 4a-ii? *

Copy and paste relevant sections from the manuscript (include quotes in quotation marks "like this" to indicate direct quotes from your manuscript), or elaborate on this item by providing additional information not in the ms, or briefly explain why the item is not applicable/relevant for your study

In methods section: "Both the protocol and recruitment methods have been previously published [22]."
In methods section: "Recruitment occurred via letters of invitation from participating General Practitioners (GP) (Primary Care Physician) in two Medicare Locals (Australian primary health care services unit responsible for coordinating care over a specified geographic area) or via electronic or print advertisements including Facebook and Google (social media and advertising), university electronic newsletters, printed posters, mailbox drops and newspapers. Young adults were compensated for their participation by receiving gift vouchers for completing 12-week online surveys and attending an in-person weigh-in."

4a-iii) Information giving during recruitment

Information given during recruitment. Specify how participants were briefed for recruitment and in the informed consent procedures (e.g., publish the informed consent documentation as appendix, see also item X26), as this information may have an effect on user self-selection, user expectation and may also bias results.

	1	2	3	4	5	
subitem not at all important	○	○	○	○	◉	essential

Does your paper address subitem 4a-iii?

Copy and paste relevant sections from the manuscript (include quotes in quotation marks "like this" to indicate direct quotes from your manuscript), or elaborate on this item by providing additional information not in the ms, or briefly explain why the item is not applicable/relevant for your study

In methods section: "Both the protocol and recruitment methods have been previously published [22]."
In methods section: "consent form and study information statement and contact information (Multimedia Appendix 1)."

4b) Settings and locations where the data were collected

Does your paper address CONSORT subitem 4b? *

Copy and paste relevant sections from the manuscript (include quotes in quotation marks "like this" to indicate direct quotes from your manuscript), or elaborate on this item by providing additional information not in the ms, or briefly explain why the item is not applicable/relevant for your study

In methods section: "Body weight (kg) and height (cm) data was collected to calculate BMI (kg m-2) at baseline via both measured and self-report methods. Participant’s GP used a standardized protocol to measure body weight to the nearest 0.1 kg and height to the nearest 0.1 cm at baseline [25]. Participants in both arms were invited for an optional in-person body weight (kg) and height (kg) at the University Metabolic Facility within a two week window following the 12-week intervention completion (weeks 13 to 14).
Online surveys were administered at baseline and within a two-week window following the 12-week intervention completion. All data were reported by participants via an online de-identified survey website (Survey Monkey®) from which data was downloaded for analysis."

4b-i) Report if outcomes were (self-)assessed through online questionnaires

Clearly report if outcomes were (self-)assessed through online questionnaires (as common in web-based trials) or otherwise.

	1	2	3	4	5	
subitem not at all important	○	○	○	○	◉	essential

Does your paper address subitem 4b-i? *

Copy and paste relevant sections from the manuscript (include quotes in quotation marks "like this" to indicate direct quotes from your manuscript), or elaborate on this item by providing additional information not in the ms, or briefly explain why the item is not applicable/relevant for your study

In methods section: "Body weight (kg) and height (cm) data was collected to calculate BMI (kg m-2) at baseline via both measured and self-report methods. Participant’s GP used a standardized protocol to measure body weight to the nearest 0.1 kg and height to the nearest 0.1 cm at baseline [25]. Participants in both arms were invited for an optional in-person body weight (kg) and height (kg) at the University Metabolic Facility within a two week window following the 12-week intervention completion (weeks 13 to 14).
Online surveys were administered at baseline and within a two-week window following the 12-week intervention completion. All data were reported by participants via an online de-identified survey website (Survey Monkey®) from which data was downloaded for analysis."

4b-ii) Report how institutional affiliations are displayed

Report how institutional affiliations are displayed to potential participants [on ehealth media], as affiliations with prestigious hospitals or universities may affect volunteer rates, use, and reactions with regards to an intervention.(Not a required item – describe only if this may bias results)

	1	2	3	4	5	
subitem not at all important	○	○	○	○	○	essential

Does your paper address subitem 4b-ii?

Copy and paste relevant sections from the manuscript (include quotes in quotation marks "like this" to indicate direct quotes from your manuscript), or elaborate on this item by providing additional

information not in the ms, or briefly explain why the item is not applicable/relevant for your study

Not applicable.

5) The interventions for each group with sufficient details to allow replication, including how and when they were actually administered

5-i) Mention names, credential, affiliations of the developers, sponsors, and owners

Mention names, credential, affiliations of the developers, sponsors, and owners [6] (if authors/evaluators are owners or developer of the software, this needs to be declared in a “Conflict of interest” section or mentioned elsewhere in the manuscript).

	1	2	3	4	5	
subitem not at all important	○	○	○	○	●	essential

Does your paper address subitem 5-i?

Copy and paste relevant sections from the manuscript (include quotes in quotation marks "like this" to indicate direct quotes from your manuscript), or elaborate on this item by providing additional information not in the ms, or briefly explain why the item is not applicable/relevant for your study

In methods section: "All study material was designed specifically for the use in the study only."

5-ii) Describe the history/development process

Describe the history/development process of the application and previous formative evaluations (e.g., focus groups, usability testing), as these will have an impact on adoption/use rates and help with interpreting results.

	1	2	3	4	5	
subitem not at all important	○	○	○	○	●	essential

Does your paper address subitem 5-ii?

Copy and paste relevant sections from the manuscript (include quotes in quotation marks "like this" to indicate direct quotes from your manuscript), or elaborate on this item by providing additional information not in the ms, or briefly explain why the item is not applicable/relevant for your study

In methods section with references provided: "and access to purpose-designed Smartphone applications that provided education and allowed self-monitoring [30]; community blog and support resources available on a website designed for the study [31]."

5-iii) Revisions and updating

Revisions and updating. Clearly mention the date and/or version number of the application/intervention (and comparator, if applicable) evaluated, or describe whether the intervention underwent major changes during the evaluation process, or whether the development and/or content was “frozen” during the trial. Describe dynamic components such as news feeds or changing content which may have an impact on the replicability of the intervention (for unexpected events see item 3b).

	1	2	3	4	5	
subitem not at all important	○	○	○	○	●	essential

Does your paper address subitem 5-iii?

Copy and paste relevant sections from the manuscript (include quotes in quotation marks "like this" to indicate direct quotes from your manuscript), or elaborate on this item by providing additional information not in the ms, or briefly explain why the item is not applicable/relevant for your study

Not applicable. No Revisions and updating.

5-iv) Quality assurance methods

Provide information on quality assurance methods to ensure accuracy and quality of information provided [1], if applicable.

	1	2	3	4	5	
subitem not at all important	○	○	●	○	○	essential

Does your paper address subitem 5-iv?

Copy and paste relevant sections from the manuscript (include quotes in quotation marks "like this" to indicate direct quotes from your manuscript), or elaborate on this item by providing additional information not in the ms, or briefly explain why the item is not applicable/relevant for your study

In methods section: "We also compared baseline characteristics and baseline primary or secondary outcomes between completers and non-completers and in-person weigh-in attenders and non-attenders using Chi-squared tests for categorical variables and independent sample T-Tests for continuous variables. We compared self-reported weight, and BMI, with measured values using paired t-tests. Analyses were performed using SPSS Version 22.0 (IBM Corp, Armonk, NY, USA), Stata Statistical Software Release 13 (Stata Corp, College Station, TX, USA) and SAS Version 9.2 (SAS Institute Inc., Cary, NC, USA) on the full intention-to-treat sample."
In results section: "At baseline there was no significant difference between measured and self-reported weight and BMI (248/250) (Ps > 0.11). At 12-weeks among participants with a measured weight (124/250), average self-reported weight was 0.7 kg (± 1.3 SD) less than the measured weight (P < 0.001). However, there was no difference between intervention (56/125; 0.8 kg ± 1.2 SD) and control groups (68/125; 0.6 kg ± 1.4 SD) (P = 0.44). There was no difference between measured and self-reported BMI at 12-weeks (P = 0.26)."

5-v) Ensure replicability by publishing the source code, and/or providing screenshots/screen-capture video, and/or providing flowcharts of the algorithms used

Ensure replicability by publishing the source code, and/or providing screenshots/screen-capture video, and/or providing flowcharts of the algorithms used. Replicability (i.e., other researchers should in principle be able to replicate the study) is a hallmark of scientific reporting.

	1	2	3	4	5	
subitem not at all important	○	○	●	○	○	essential

Does your paper address subitem 5-v?

Copy and paste relevant sections from the manuscript (include quotes in quotation marks "like this" to indicate direct quotes from your manuscript), or elaborate on this item by providing additional information not in the ms, or briefly explain why the item is not applicable/relevant for your study

Not applicable.

5-vi) Digital preservation

Digital preservation: Provide the URL of the application, but as the intervention is likely to change or disappear over the course of the years; also make sure the intervention is archived (Internet Archive, webcitation.org, and/or publishing the source code or screenshots/videos alongside the article). As pages behind login screens cannot be archived, consider creating demo pages which are accessible without login.

	1	2	3	4	5	
subitem not at all important	○	○	○	●	○	essential

Does your paper address subitem 5-vi?

Copy and paste relevant sections from the manuscript (include quotes in quotation marks "like this" to indicate direct quotes from your manuscript), or elaborate on this item by providing additional information not in the ms, or briefly explain why the item is not applicable/relevant for your study

In methods section: "website designed for the study [31]". More access will be created once all program components are complete.

5-vii) Access

Access: Describe how participants accessed the application, in what setting/context, if they had to pay (or were paid) or not, whether they had to be a member of specific group. If known, describe how participants obtained “access to the platform and Internet” [1]. To ensure access for editors/reviewers/readers, consider to provide a “backdoor” login account or demo mode for reviewers/readers to explore the application (also important for archiving purposes, see vi).

	1	2	3	4	5	
subitem not at all important	○	○	○	◉	○	essential

Does your paper address subitem 5-vii? *

Copy and paste relevant sections from the manuscript (include quotes in quotation marks "like this" to indicate direct quotes from your manuscript), or elaborate on this item by providing additional information not in the ms, or briefly explain why the item is not applicable/relevant for your study

In methods section "password-protected access to purpose-designed Smartphone applications that provided education and allowed self-monitoring [30]; community blog and support resources available on a password-protected website designed for the study [31]."

5-viii) Mode of delivery, features/functionalities/components of the intervention and comparator, and the theoretical framework

Describe mode of delivery, features/functionalities/components of the intervention and comparator, and the theoretical framework [6] used to design them (instructional strategy [1], behaviour change techniques, persuasive features, etc., see e.g., [7, 8] for terminology). This includes an in-depth description of the content (including where it is coming from and who developed it) [1],” whether [and how] it is tailored to individual circumstances and allows users to track their progress and receive feedback” [6]. This also includes a description of communication delivery channels and – if computer-mediated communication is a component – whether communication was synchronous or asynchronous [6]. It also includes information on presentation strategies [1], including page design principles, average amount of text on pages, presence of hyperlinks to other resources, etc. [1].

	1	2	3	4	5	
subitem not at all important	○	○	○	◉	○	essential

Does your paper address subitem 5-viii? *

Copy and paste relevant sections from the manuscript (include quotes in quotation marks "like this" to indicate direct quotes from your manuscript), or elaborate on this item by providing additional information not in the ms, or briefly explain why the item is not applicable/relevant for your study

Described in protocol paper. Reader is referred in methods section.

5-ix) Describe use parameters

Describe use parameters (e.g., intended “doses” and optimal timing for use). Clarify what instructions or recommendations were given to the user, e.g., regarding timing, frequency, heaviness of use, if any, or was the intervention used ad libitum.

	1	2	3	4	5	
subitem not at all important	○	○	○	○	●	essential

Does your paper address subitem 5-ix?

Copy and paste relevant sections from the manuscript (include quotes in quotation marks "like this" to indicate direct quotes from your manuscript), or elaborate on this item by providing additional information not in the ms, or briefly explain why the item is not applicable/relevant for your study

In methods section: "Engagement with the intervention was assessed using text message replies and number of coaching calls completed. Intervention participants were asked to reply ‘OK’ to 16 messages in the 12-weeks and control participants were asked to reply ‘OK’ to all four text messages. Text messages delivery reports were created from the text message service provider (My Message Media® program) for delivery status and replies. Detailed records of all coaching calls were collated in a database. The 12-week post-intervention survey also asked participants their access to and use of program materials."

5-x) Clarify the level of human involvement

Clarify the level of human involvement (care providers or health professionals, also technical assistance) in the e-intervention or as co-intervention (detail number and expertise of professionals involved, if any, as well as “type of assistance offered, the timing and frequency of the support, how it is initiated, and the medium by which the assistance is delivered”. It may be necessary to distinguish between the level of human involvement required for the trial, and the level of human involvement required for a routine application outside of a RCT setting (discuss under item 21 – generalizability).

	1	2	3	4	5	
subitem not at all important	○	○	○	●	○	essential

Does your paper address subitem 5-x?

Copy and paste relevant sections from the manuscript (include quotes in quotation marks "like this" to indicate direct quotes from your manuscript), or elaborate on this item by providing additional

information not in the ms, or briefly explain why the item is not applicable/relevant for your study

In the methods section: "five personalized coaching calls" "Two Accredited Practising Dietitians conducted the coaching calls according to a standardized protocol and allowed the participants to set goals, discuss barriers and enablers and their progress."

5-xi) Report any prompts/reminders used

Report any prompts/reminders used: Clarify if there were prompts (letters, emails, phone calls, SMS) to use the application, what triggered them, frequency etc. It may be necessary to distinguish between the level of prompts/reminders required for the trial, and the level of prompts/reminders for a routine application outside of a RCT setting (discuss under item 21 – generalizability).

	1	2	3	4	5	
subitem not at all important	○	○	○	●	○	essential

Does your paper address subitem 5-xi? *

Copy and paste relevant sections from the manuscript (include quotes in quotation marks "like this" to indicate direct quotes from your manuscript), or elaborate on this item by providing additional information not in the ms, or briefly explain why the item is not applicable/relevant for your study

In methods section: "One email was sent each week reiterating the information in the text messages and included links to the Smartphone applications to remind participants."

5-xii) Describe any co-interventions (incl. training/support)

Describe any co-interventions (incl. training/support): Clearly state any interventions that are provided in addition to the targeted eHealth intervention, as ehealth intervention may not be designed as stand-alone intervention. This includes training sessions and support [1]. It may be necessary to distinguish between the level of training required for the trial, and the level of training for a routine application outside of a RCT setting (discuss under item 21 – generalizability.

	1	2	3	4	5	
subitem not at all important	○	○	○	○	○	essential

Does your paper address subitem 5-xii? *

Copy and paste relevant sections from the manuscript (include quotes in quotation marks "like this" to indicate direct quotes from your manuscript), or elaborate on this item by providing additional information not in the ms, or briefly explain why the item is not applicable/relevant for your study

Not applicable. No co-interventions.

6a) Completely defined pre-specified primary and secondary outcome measures, including how and when they were assessed

Does your paper address CONSORT subitem 6a? *

Copy and paste relevant sections from the manuscript (include quotes in quotation marks "like this" to indicate direct quotes from your manuscript), or elaborate on this item by providing additional information not in the ms, or briefly explain why the item is not applicable/relevant for your study

In methods section: "Demographic characteristics were collected by online survey and included age, gender, postcode (for categorizing socio-economic status [23]), ethnicity (language spoken at home [24]), education level [24] and income ($AU) [24]. Body weight (kg) and height (cm) data was collected to calculate BMI (kg m-2) at baseline via both measured and self-report methods. Participant's GP used a standardized protocol to measure body weight to the nearest 0.1 kg and height to the nearest 0.1 cm at baseline [25]. Participants in both arms were invited for an optional in-person body weight (kg) and height (kg) at the University Metabolic Facility within a two week window following the 12-week intervention completion (weeks 13 to 14). Measures were taken by two higher degree research students blinded to participant allocation.
Online surveys were administered at baseline and within a two-week window following the 12-week intervention completion (weeks 13 to 14). Data collected included self-reported weight (kg) and height (cm); short categorical questions to assess usual weekly intake of SSB [26] and daily intake of fruit and vegetables [26] and weekly take-away meals [27]; questions about physical activity in the previous seven days using the short form International Physical Activity Questionnaire (IPAQ) [28]. IPAQ was scored using established methods [29] and data reported as a continuous measure in metabolic equivalence of task (MET)-minutes per week. All data were reported by participants via an online de-identified survey website (Survey Monkey®) from which data was downloaded for analysis.
Engagement with the intervention was assessed using text message replies and number of coaching calls completed. Intervention participants were asked to reply 'OK' to 16 messages in the 12-weeks and control participants were asked to reply 'OK' to all four text messages. Text messages delivery reports were created from the text message service provider (My Message Media® program) for delivery status and replies. Detailed records of all coaching calls were collated in a database. The 12-week post-intervention survey also asked participants their access to and use of program materials."

6a-i) Online questionnaires: describe if they were validated for online use and apply CHERRIES items to describe how the questionnaires were designed/deployed

If outcomes were obtained through online questionnaires, describe if they were validated for online use and apply CHERRIES items to describe how the questionnaires were designed/deployed [9].

	1	2	3	4	5	
subitem not at all important	○	○	○	○	◉	essential

Does your paper address subitem 6a-i?

Copy and paste relevant sections from manuscript text

Described in protocol paper. Reader is referred in methods section.

6a-ii) Describe whether and how “use” (including intensity of use/dosage) was defined/measured/monitored

Describe whether and how “use” (including intensity of use/dosage) was defined/measured/monitored (logins, logfile analysis, etc.). Use/adoption metrics are important process outcomes that should be reported in any ehealth trial.

	1	2	3	4	5	
subitem not at all important	○	○	○	○	◉	essential

Does your paper address subitem 6a-ii?

Copy and paste relevant sections from manuscript text

In methods section: "Engagement with the intervention was assessed using text message replies and number of coaching calls completed. Intervention participants were asked to reply ‘OK’ to 16 messages in the 12-weeks and control participants were asked to reply ‘OK’ to all four text messages. Text messages delivery reports were created from the text message service provider (My Message Media® program) for delivery status and replies. Detailed records of all coaching calls were collated in a database. The 12-week post-intervention survey also asked participants their access to and use of program materials."

6a-iii) Describe whether, how, and when qualitative feedback from participants was obtained

Describe whether, how, and when qualitative feedback from participants was obtained (e.g., through emails, feedback forms, interviews, focus groups).

1 2 3 4 5

subitem not at all important ○ ○ ○ ○ ◉ essential

Does your paper address subitem 6a-iii?

Copy and paste relevant sections from manuscript text

Qualitative process evaluation currently in progress and will form the basis of another manuscript.

6b) Any changes to trial outcomes after the trial commenced, with reasons

Does your paper address CONSORT subitem 6b? *

Copy and paste relevant sections from the manuscript (include quotes in quotation marks "like this" to indicate direct quotes from your manuscript), or elaborate on this item by providing additional information not in the ms, or briefly explain why the item is not applicable/relevant for your study

Not applicable. No Any changes to trial outcomes after the trial commenced.

7a) How sample size was determined

NPT: When applicable, details of whether and how the clustering by care provides or centers was addressed

7a-i) Describe whether and how expected attrition was taken into account when calculating the sample size

Describe whether and how expected attrition was taken into account when calculating the sample size.

1 2 3 4 5

subitem not at all important ○ ○ ○ ◉ ○ essential

Does your paper address subitem 7a-i?

Copy and paste relevant sections from manuscript title (include quotes in quotation marks "like this" to indicate direct quotes from your manuscript), or elaborate on this item by providing additional information not in the ms, or briefly explain why the item is not applicable/relevant for your study

In methods section: "Based on our previous meta-analysis [6], it appeared a difference of 1.7 kg could be expected. The sample size required for detection of a difference of 2 kg with 80% power, significance level of 0.05, 10 kg standard deviation and a correlation between baseline and final weight of 0.8 was 354 subjects after allowing for a 20 % drop-out. Due to slower than expected recruitment rate and with time and funding constraints, recruitment was stopped at 250 participants."

7b) When applicable, explanation of any interim analyses and stopping guidelines

Does your paper address CONSORT subitem 7b? *

Copy and paste relevant sections from the manuscript (include quotes in quotation marks "like this" to indicate direct quotes from your manuscript), or elaborate on this item by providing additional information not in the ms, or briefly explain why the item is not applicable/relevant for your study

In methods section: "Due to slower than expected recruitment rate and with time and funding constraints, recruitment was stopped at 250 participants."

8a) Method used to generate the random allocation sequence

NPT: When applicable, how care providers were allocated to each trial group

Does your paper address CONSORT subitem 8a? *

Copy and paste relevant sections from the manuscript (include quotes in quotation marks "like this" to indicate direct quotes from your manuscript), or elaborate on this item by providing additional information not in the ms, or briefly explain why the item is not applicable/relevant for your study

In methods section: "A random sequence was generated by an independent researcher and concealed from those responsible for enrolling participants into the intervention arm. Eligible participants were randomised in a 1:1 ratio into intervention and control arms. Randomization was based on a stratified randomised block design, where the strata were the GP clinic and participant gender. While participants were aware of another arm to the trial, every attempt was made to ensure that the nature of this other arm was not revealed."

8b) Type of randomisation; details of any restriction (such as blocking and block size)

Does your paper address CONSORT subitem 8b? *

Copy and paste relevant sections from the manuscript (include quotes in quotation marks "like this" to indicate direct quotes from your manuscript), or elaborate on this item by providing additional information not in the ms, or briefly explain why the item is not applicable/relevant for your study

In methods section: "A random sequence was generated by an independent researcher and concealed from those responsible for enrolling participants into the intervention arm. Eligible participants were randomised in a 1:1 ratio into intervention and control arms. Randomization was based on a stratified randomised block design, where the strata were the GP clinic and participant gender. While participants were aware of another arm to the trial, every attempt was made to ensure that the nature of this other arm was not revealed."

9) Mechanism used to implement the random allocation sequence (such as sequentially numbered containers), describing any steps taken to conceal the sequence until interventions were assigned

Does your paper address CONSORT subitem 9? *

Copy and paste relevant sections from the manuscript (include quotes in quotation marks "like this" to indicate direct quotes from your manuscript), or elaborate on this item by providing additional information not in the ms, or briefly explain why the item is not applicable/relevant for your study

In methods section: "A random sequence was generated by an independent researcher and concealed from those responsible for enrolling participants into the intervention arm. Eligible participants were randomised in a 1:1 ratio into intervention and control arms. Randomization was based on a stratified randomised block design, where the strata were the GP clinic and participant gender. While participants were aware of another arm to the trial, every attempt was made to ensure that the nature of this other arm was not revealed."

10) Who generated the random allocation sequence, who enrolled participants, and who assigned participants to interventions

Does your paper address CONSORT subitem 10? *

Copy and paste relevant sections from the manuscript (include quotes in quotation marks "like this" to indicate direct quotes from your manuscript), or elaborate on this item by providing additional information not in the ms, or briefly explain why the item is not applicable/relevant for your study

In methods section: "A random sequence was generated by an independent researcher and concealed from those responsible for enrolling participants into the intervention arm. Eligible participants were randomised in a 1:1 ratio into intervention and control arms. Randomization was based on a stratified randomised block design, where the strata were the GP clinic and participant gender. While participants were aware of another arm to the trial, every attempt was made to ensure that the nature of this other arm was not revealed."

11a) If done, who was blinded after assignment to interventions (for example, participants, care providers, those assessing outcomes) and how

NPT: Whether or not administering co-interventions were blinded to group assignment

11a-i) Specify who was blinded, and who wasn’t

Specify who was blinded, and who wasn’t. Usually, in web-based trials it is not possible to blind the participants [1, 3] (this should be clearly acknowledged), but it may be possible to blind outcome assessors, those doing data analysis or those administering co-interventions (if any).

	1	2	3	4	5	
subitem not at all important	○	○	○	○	◉	essential

Does your paper address subitem 11a-i? *

Copy and paste relevant sections from the manuscript (include quotes in quotation marks "like this" to indicate direct quotes from your manuscript), or elaborate on this item by providing additional information not in the ms, or briefly explain why the item is not applicable/relevant for your study

In methods section: "A random sequence was generated by an independent researcher and concealed from those responsible for enrolling participants into the intervention arm."
"While participants were aware of another arm to the trial, every attempt was made to ensure that the nature of this other arm was not revealed."
"Measures were taken by two higher degree research students blinded to participant allocation."
"Researchers analysing participant outcomes were blinded to participant allocation."

11a-ii) Discuss e.g., whether participants knew which intervention was the “intervention of interest” and which one was the “comparator”

Informed consent procedures (4a-ii) can create biases and certain expectations - discuss e.g., whether participants knew which intervention was the “intervention of interest” and which one was the “comparator”.

	1	2	3	4	5	
subitem not at all important	○	○	○	○	○	essential

Does your paper address subitem 11a-ii?

Copy and paste relevant sections from the manuscript (include quotes in quotation marks "like this" to indicate direct quotes from your manuscript), or elaborate on this item by providing additional information not in the ms, or briefly explain why the item is not applicable/relevant for your study

In methods section: "While participants were aware of another arm to the trial, every attempt was made to ensure that the nature of this other arm was not revealed."

11b) If relevant, description of the similarity of interventions

(this item is usually not relevant for ehealth trials as it refers to similarity of a placebo or sham intervention to a active medication/intervention)

Does your paper address CONSORT subitem 11b? *

Copy and paste relevant sections from the manuscript (include quotes in quotation marks "like this" to indicate direct quotes from your manuscript), or elaborate on this item by providing additional information not in the ms, or briefly explain why the item is not applicable/relevant for your study

Intervention vs. control group delivery compared in methods section.

12a) Statistical methods used to compare groups for primary and secondary outcomes

NPT: When applicable, details of whether and how the clustering by care providers or centers was addressed

Does your paper address CONSORT subitem 12a? *

Copy and paste relevant sections from the manuscript (include quotes in quotation marks "like this" to indicate direct quotes from your manuscript), or elaborate on this item by providing additional information not in the ms, or briefly explain why the item is not applicable/relevant for your study

In methods section: "The primary outcomes, body weight (kg) and BMI (kg m-2) at 12-weeks were compared between the two groups using analysis-of-covariance models adjusting for baseline values, GP clinic and gender. Secondary outcomes that were continuous (physical activity MET-minutes and physical activity days) were also analysed using analysis-of-covariance models. Robust regression models were used for analyses where residuals indicated non-Normality. Secondary outcomes that were categorical (fruit and vegetable servings per day; SSB consumption per week and energy-dense take away meal intake per week) were analysed using Mantel-Haenszel Chi-Square tests stratified by GP clinic and gender. The analysis used the 'intention-to-treat' principle with multiple imputations to account for missing data. Five imputed datasets were created and the results for continuous outcomes pooled using Rubin's rules. Chi-square statistics were pooled, and P-values estimated, using the method described by Li et al., 1991 [35]. $P < 0.05$ was considered statistically significant. Researchers analysing participant outcomes were blinded to participant allocation.
We also compared baseline characteristics and baseline primary or secondary outcomes between completers and non-completers and in-person weigh-in attenders and non-attenders using Chi-squared tests for categorical variables and independent sample T-Tests for continuous variables. We compared self-reported weight, and BMI, with measured values using paired t-tests. Analyses were performed using SPSS Version 22.0 (IBM Corp, Armonk, NY, USA), Stata Statistical Software Release 13 (Stata Corp, College Station, TX, USA) and SAS Version 9.2 (SAS Institute Inc., Cary, NC, USA) on the full intention-to-treat sample."

12a-i) Imputation techniques to deal with attrition / missing values

Imputation techniques to deal with attrition / missing values: Not all participants will use the intervention/comparator as intended and attrition is typically high in ehealth trials. Specify how participants who did not use the application or dropped out from the trial were treated in the statistical analysis (a complete case analysis is strongly discouraged, and simple imputation techniques such as LOCF may also be problematic [4]).

	1	2	3	4	5	
subitem not at all important	○	○	○	●	○	essential

Does your paper address subitem 12a-i? *

Copy and paste relevant sections from the manuscript (include quotes in quotation marks "like this" to indicate direct quotes from your manuscript), or elaborate on this item by providing additional information not in the ms, or briefly explain why the item is not applicable/relevant for your study

In methods section: "The analysis used the ‘intention-to-treat’ principle with multiple imputations to account for missing data. Five imputed datasets were created and the results for continuous outcomes pooled using Rubin’s rules. Chi-square statistics were pooled, and P-values estimated, using the method described by Li et al., 1991 [35]."

12b) Methods for additional analyses, such as subgroup analyses and adjusted analyses

Does your paper address CONSORT subitem 12b? *

Copy and paste relevant sections from the manuscript (include quotes in quotation marks "like this" to indicate direct quotes from your manuscript), or elaborate on this item by providing additional information not in the ms, or briefly explain why the item is not applicable/relevant for your study

In methods section: "We also compared baseline characteristics and baseline primary or secondary outcomes between completers and non-completers and in-person weigh-in attenders and non-attenders using Chi-squared tests for categorical variables and independent sample T-Tests for continuous variables. We compared self-reported weight, and BMI, with measured values using paired t-tests. Analyses were performed using SPSS Version 22.0 (IBM Corp, Armonk, NY, USA), Stata Statistical Software Release 13 (Stata Corp, College Station, TX, USA) and SAS Version 9.2 (SAS Institute Inc., Cary, NC, USA) on the full intention-to-treat sample."

X26) REB/IRB Approval and Ethical Considerations [recommended as subheading under "Methods"] (not a CONSORT item)

X26-i) Comment on ethics committee approval

1 2 3 4 5

subitem not at all important ○ ○ ○ ○ ◉ essential

Does your paper address subitem X26-i?

Copy and paste relevant sections from the manuscript (include quotes in quotation marks "like this" to indicate direct quotes from your manuscript), or elaborate on this item by providing additional information not in the ms, or briefly explain why the item is not applicable/relevant for your study

In methods section: "The trial was approved by the University Human Research Ethics Committee in September 2012 (Approval Number 15226) and all the participants gave written informed consent."

x26-ii) Outline informed consent procedures

Outline informed consent procedures e.g., if consent was obtained offline or online (how? Checkbox, etc.?), and what information was provided (see 4a-ii). See [6] for some items to be included in informed consent documents.

1 2 3 4 5

subitem not at all important ○ ○ ○ ○ ○ essential

Does your paper address subitem X26-ii?

Copy and paste relevant sections from the manuscript (include quotes in quotation marks "like this" to indicate direct quotes from your manuscript), or elaborate on this item by providing additional information not in the ms, or briefly explain why the item is not applicable/relevant for your study

In methods section: "The trial was approved by the University Human Research Ethics Committee in September 2012 (Approval Number 15226) and all the participants gave written informed consent."

X26-iii) Safety and security procedures

Safety and security procedures, incl. privacy considerations, and any steps taken to reduce the likelihood or detection of harm (e.g., education and training, availability of a hotline)

1 2 3 4 5

subitem not at all important ○ ○ ○ ○ ◉ essential

Does your paper address subitem X26-iii?

Copy and paste relevant sections from the manuscript (include quotes in quotation marks "like this" to indicate direct quotes from your manuscript), or elaborate on this item by providing additional information not in the ms, or briefly explain why the item is not applicable/relevant for your study

RESULTS

13a) For each group, the numbers of participants who were randomly assigned, received intended treatment, and were analysed for the primary outcome

NPT: The number of care providers or centers performing the intervention in each group and the number of patients treated by each care provider in each center

Does your paper address CONSORT subitem 13a? *

Copy and paste relevant sections from the manuscript (include quotes in quotation marks "like this" to indicate direct quotes from your manuscript), or elaborate on this item by providing additional information not in the ms, or briefly explain why the item is not applicable/relevant for your study

Intention to treat analysis as mentioned above.
In results section: "Recruitment resulted in 1181 enquires, of which 79% (931/1181) were excluded or failed to complete screening requirements (Figure 1). Two-hundred and fifty young adults were randomly assigned to intervention or control groups. Eight participants (6.4%) dropped out from the intervention group during the 12-week intervention (one for medical reasons; two for personal reasons; two moved overseas; two found the program not suitable and one for other reasons not stated) and an additional five participants failed to complete post intervention online surveys (4.0%) in the intervention group (10.4% total) (Figure 1). No participants dropped out of the control group and 21 participants in the control group (16.8%) did not complete post intervention online surveys. Completers and non-completers did not differ significantly in allocation, baseline demographic characteristics or baseline primary or secondary outcomes (Ps > 0.11). Except non-completes consumed more takeout meals at baseline (P = 0.004). Nearly half of all participants (124/250, 49.6%) accepted the invitation for an in-person weight and height (intervention: 56/125, 44.8%; control: 68/125, 54.4%). There were no significant differences in baseline characteristics between participants that attended the in-person body weight and those that did not (Ps > 0.34), except those attending ate less fruit at baseline (P = 0.03)."

13b) For each group, losses and exclusions after randomisation, together with reasons

Does your paper address CONSORT subitem 13b? (NOTE: Preferably, this is shown in a CONSORT flow diagram) *

Copy and paste relevant sections from the manuscript (include quotes in quotation marks "like this" to indicate direct quotes from your manuscript), or elaborate on this item by providing additional information not in the ms, or briefly explain why the item is not applicable/relevant for your study

In results section: "Recruitment resulted in 1181 enquires, of which 79% (931/1181) were excluded or failed to complete screening requirements (Figure 1). Two-hundred and fifty young adults were randomly assigned to intervention or control groups. Eight participants (6.4%) dropped out from the intervention group during the 12-week intervention (one for medical reasons; two for personal reasons; two moved overseas; two found the program not suitable and one for other reasons not stated) and an additional five participants failed to complete post intervention online surveys (4.0%) in the intervention group (10.4% total) (Figure 1). No participants dropped out of the control group and 21 participants in the control group (16.8%) did not complete post intervention online surveys. Completers and non-completers did not differ significantly in allocation, baseline demographic characteristics or baseline primary or secondary outcomes (Ps > 0.11). Except non-completes consumed more takeout meals at baseline (P = 0.004). Nearly half of all participants (124/250, 49.6%) accepted the invitation for an in-person weight and height (intervention: 56/125, 44.8%; control: 68/125, 54.4%). There were no significant differences in baseline characteristics between participants that attended the in-person body weight and those that did not (Ps > 0.34), except those attending ate less fruit at baseline (P = 0.03)."

13b-i) Attrition diagram

Strongly recommended: An attrition diagram (e.g., proportion of participants still logging in or using the intervention/comparator in each group plotted over time, similar to a survival curve) or other figures or tables demonstrating usage/dose/engagement.

	1	2	3	4	5	
subitem not at all important	○	○	○	○	●	essential

Does your paper address subitem 13b-i?

Copy and paste relevant sections from the manuscript or cite the figure number if applicable (include quotes in quotation marks "like this" to indicate direct quotes from your manuscript), or elaborate on this item by providing additional information not in the ms, or briefly explain why the item is not applicable/relevant for your study

Please refer to figure 1.

14a) Dates defining the periods of recruitment and follow-up

Does your paper address CONSORT subitem 14a? *

Copy and paste relevant sections from the manuscript (include quotes in quotation marks "like this" to indicate direct quotes from your manuscript), or elaborate on this item by providing additional information not in the ms, or briefly explain why the item is not applicable/relevant for your study

In methods section: "The TXT2BFiT study is a two arm parallel design RCT in 18 to 35 year olds recruited from the Greater Sydney Area NSW, Australia between November, 2012 and July, 2014."

14a-i) Indicate if critical “secular events” fell into the study period

Indicate if critical “secular events” fell into the study period, e.g., significant changes in Internet resources available or “changes in computer hardware or Internet delivery resources”

	1	2	3	4	5	
subitem not at all important	○	○	◉	○	○	essential

Does your paper address subitem 14a-i?

Copy and paste relevant sections from the manuscript (include quotes in quotation marks "like this" to indicate direct quotes from your manuscript), or elaborate on this item by providing additional information not in the ms, or briefly explain why the item is not applicable/relevant for your study

Not applicable. No critical “secular events” fell into the study period.

14b) Why the trial ended or was stopped (early)

Does your paper address CONSORT subitem 14b? *

Copy and paste relevant sections from the manuscript (include quotes in quotation marks "like this" to indicate direct quotes from your manuscript), or elaborate on this item by providing additional information not in the ms, or briefly explain why the item is not applicable/relevant for your study

In methods section: "Due to slower than expected recruitment rate and with time and funding constraints, recruitment was stopped at 250 participants."

15) A table showing baseline demographic and clinical characteristics for each group

NPT: When applicable, a description of care providers (case volume, qualification, expertise, etc.) and centers (volume) in each group

Does your paper address CONSORT subitem 15? *

Copy and paste relevant sections from the manuscript (include quotes in quotation marks "like this" to indicate direct quotes from your manuscript), or elaborate on this item by providing additional information not in the ms, or briefly explain why the item is not applicable/relevant for your study

Please refer to table 1.
In results section: "Baseline characteristics of participants are shown in Table 1. The total randomised sample were mostly older (43% 30 years or older), female (61%), English speaking only (69%), highly educated and living in a socio-economically advantaged area (61% and 76% respectively). Participants were overweight on the basis of BMI classification (intervention 27.3 kg m -2; control 27.1 kg m -2) (Table 2 and Table 3). Table 4 shows that by design, most participants did not meet the recommended serves of fruit (intervention 66.7%; control 61.6%) or serves of vegetables (intervention 94.3%; control 96.8%) [34]; consumed SSB (greater than one litre per week for intervention 13.0%; control 17.6%) and participants consumed two or more take-away meals per week (intervention 61.0%; control 63.2%). All participants reported above average levels of recommended physical activity [36] (intervention 1619.9 MET-minutes per week; control 1646.8 MET-minutes per week) (Table 5)."

15-i) Report demographics associated with digital divide issues

In ehealth trials it is particularly important to report demographics associated with digital divide issues, such as age, education, gender, social-economic status, computer/Internet/ehealth literacy of the participants, if known.

	1	2	3	4	5	
subitem not at all important	○	○	○	◉	○	essential

Does your paper address subitem 15-i? *

Copy and paste relevant sections from the manuscript (include quotes in quotation marks "like this" to indicate direct quotes from your manuscript), or elaborate on this item by providing additional information not in the ms, or briefly explain why the item is not applicable/relevant for your study

Please refer to table 1.
In results section: "Baseline characteristics of participants are shown in Table 1. The total randomised sample were mostly older (43% 30 years or older), female (61%), English speaking only (69%), highly educated and living in a socio-economically advantaged area (61% and 76% respectively). Participants were overweight on the basis of BMI classification (intervention 27.3 kg m -2; control 27.1 kg m -2) (Table 2 and Table 3). Table 4 shows that by design, most participants did not meet the recommended serves of fruit (intervention 66.7%; control 61.6%) or serves of vegetables (intervention 94.3%; control 96.8%) [34]; consumed SSB (greater than one litre per week for intervention 13.0%; control 17.6%) and participants consumed two or more take-away meals per week (intervention 61.0%; control 63.2%). All participants reported above average levels of recommended physical activity [36] (intervention 1619.9 MET-minutes per week; control 1646.8 MET-minutes per week) (Table 5)."

16) For each group, number of participants (denominator) included in each analysis and whether the analysis was by original assigned groups

16-i) Report multiple “denominators” and provide definitions

Report multiple “denominators” and provide definitions: Report N’s (and effect sizes) “across a range of study participation [and use] thresholds” [1], e.g., N exposed, N consented, N used more than x times, N used more than y weeks, N participants “used” the intervention/comparator at specific pre-defined time points of interest (in absolute and relative numbers per group). Always clearly define “use” of the intervention.

	1	2	3	4	5	
subitem not at all important	○	○	○	○	◉	essential

Does your paper address subitem 16-i? *

Copy and paste relevant sections from the manuscript (include quotes in quotation marks "like this" to indicate direct quotes from your manuscript), or elaborate on this item by providing additional information not in the ms, or briefly explain why the item is not applicable/relevant for your study

As previously mentioned, intention to treat analysis.

16-ii) Primary analysis should be intent-to-treat

Primary analysis should be intent-to-treat, secondary analyses could include comparing only “users”, with the appropriate caveats that this is no longer a randomized sample (see 18-i).

1	2	3	4	5

subitem not at all important ○ ○ ○ ○ ◉ essential

Does your paper address subitem 16-ii?

Copy and paste relevant sections from the manuscript (include quotes in quotation marks "like this" to indicate direct quotes from your manuscript), or elaborate on this item by providing additional information not in the ms, or briefly explain why the item is not applicable/relevant for your study

As previously mentioned, intention to treat analysis.

17a) For each primary and secondary outcome, results for each group, and the estimated effect size and its precision (such as 95% confidence interval)

Does your paper address CONSORT subitem 17a? *

Copy and paste relevant sections from the manuscript (include quotes in quotation marks "like this" to indicate direct quotes from your manuscript), or elaborate on this item by providing additional information not in the ms, or briefly explain why the item is not applicable/relevant for your study

Please refer to results section, and tables 2-5.
In results section: "Body weight (kg) and BMI (kg m-2)
Young adults in the intervention group were 2.2 kg lighter at 12-weeks compared to the control group using measured body weight after adjusting for baseline measured body weight (95% confidence intervals (CI) 0.8, 3.6; P = 0.005) (Table 2). A similar pattern was observed with BMI, which was 0.5 kg m-2 less at 12-weeks (95% CI 0.1, 1.0; P = 0.024) for the intervention group compared to the control group using measured BMI.
Using self-reported body weight measures, intervention participants were 2.1 kg (95% CI 1.4, 2.8, P < 0.001) and 0.6 BMI units (kg m-2) (95% CI 0.3, 1.0, P < 0.001) lighter than control participants at 12-weeks (Table 3).
At baseline there was no significant difference between measured and self-reported weight and BMI (248/250) (Ps > 0.11). At 12-weeks among participants with a measured weight (124/250), average self-reported weight was 0.7 kg (± 1.3 SD) less than the measured weight (P < 0.001). However, there was no difference between intervention (56/125; 0.8 kg ± 1.2 SD) and control groups (68/125; 0.6 kg ± 1.4 SD) (P = 0.44). There was no difference between measured and self-reported BMI at 12-weeks (P = 0.26).
Fruit and vegetable intake
The majority of participants reported consuming the recommended two serves of fruit per day or more after 12-weeks (Table 4), with a non-significant difference between intervention group compared to the control group (P = 0.18). Intervention participants were more likely to consume greater quantities of vegetables after 12-weeks compared to control participants (P = 0.009). For example, 35.0% of intervention participants consumed ≥ 4 serves of vegetables compared to 22.4% of control participants.
Sugar sweetened beverage and Take-out meal intake
Intervention participants consumed SSB less frequently after 12-weeks compared with the control participants (P = 0.002) (Table 4). For example, 92.7% of intervention participants consumed ≤ 500 ml of SSB compared to 72.0% of control participants at 12-weeks.
After 12 weeks, intervention participants reported consuming energy-dense take away meals less frequently during the week compared with the control participants (consuming ≤ 1 energy-dense takeaway: 88/123; 71.5% of intervention participants vs. 68/125; 54.4% of control participants) (P = 0.014) (Table 4).
Physical activity
Intervention participants reported a mean increase of 563.1 (±1983.6 SD) MET-minutes/week after 12-weeks. Control participants reported a mean increase of 244.4 (±1510.6 SD) MET-minutes/week (Table 5). These observed increases in energy expenditure were predominantly due to increased reported vigorous and walking activities, which increased by an average 243.0 (±1073.3 SD) and 231.8 (±1313.9 SD) MET-minutes/week among intervention participants respectively and 102.5 (±1148.6 SD) and 148.1 (±747.3 SD) MET- minutes/week among control participants respectively. After adjusting for baseline MET- minutes/week, GP clinic and gender there was a significant effect of the intervention on average MET- minutes/week at 12-weeks (95% CI -503.8,-1.2, P = 0.05;). Total and walking physical activity days increased more in the intervention group compared to the control group (95% CI -2.2, -0.5; P = 0.003; and 95% CI -1.1, -0.1; P = 0.02, respectively). "

17a-i) Presentation of process outcomes such as metrics of use and intensity of use

In addition to primary/secondary (clinical) outcomes, the presentation of process outcomes such as metrics of use and intensity of use (dose, exposure) and their operational definitions is critical. This

does not only refer to metrics of attrition (13-b) (often a binary variable), but also to more continuous exposure metrics such as “average session length”. These must be accompanied by a technical description how a metric like a “session” is defined (e.g., timeout after idle time) [1] (report under item 6a).

	1	2	3	4	5	
subitem not at all important	○	○	○	○	○	essential

Does your paper address subitem 17a-i?

Copy and paste relevant sections from the manuscript (include quotes in quotation marks "like this" to indicate direct quotes from your manuscript), or elaborate on this item by providing additional information not in the ms, or briefly explain why the item is not applicable/relevant for your study

Process evaluation is currently in progress.
In results section: "The mean number of coaching calls completed in the intervention group was 4.9 out of five (97.0% overall completed all five). All participants who completed the post-intervention survey reported engaging with coaching calls. Of the 12308 text messages sent during the 12-week intervention (control: 500; intervention 11808), only 2.3% were not delivered (control: 15/500, 3%; intervention 265/11808, 2.2%). Over half (66/123, 52.8%) of intervention participants replied to eight or more of the 16 SMS with a requested response, with 25/123 (20.3%) replying to all. Most control participants replied to two or more of the four SMS (114/123, 91.2%), with 62.4% (78/125) replying to all four SMS. One-hundred of the 110 (90.9%) intervention participants who completed the follow-up survey self-reported that they used the SMS messages. Email delivery was 100%; with 84/110 (76.4%) participants reporting that they used the e-mail messages during the study. Eighty-two (74.5%) intervention participants reported that they did not access the smartphone applications during the study. The mailed booklet was used by 72/110 (65.5%) of intervention participants and only 7/110 (6.4%) used the blog. Most intervention participants (65/110, 59.1%) did not use the resources available on the website. Of those that did, the take-away meal planner was reported as most used by the intervention participants (28/110, 25.5%)."

17b) For binary outcomes, presentation of both absolute and relative effect sizes is recommended

Does your paper address CONSORT subitem 17b? *

Copy and paste relevant sections from the manuscript (include quotes in quotation marks "like this" to indicate direct quotes from your manuscript), or elaborate on this item by providing additional information not in the ms, or briefly explain why the item is not applicable/relevant for your study

Not applicable. No binary outcomes.

18) Results of any other analyses performed, including subgroup analyses and adjusted analyses, distinguishing pre-specified from exploratory

Does your paper address CONSORT subitem 18? *

Copy and paste relevant sections from the manuscript (include quotes in quotation marks "like this" to indicate direct quotes from your manuscript), or elaborate on this item by providing additional information not in the ms, or briefly explain why the item is not applicable/relevant for your study

In results section: "At baseline there was no significant difference between measured and self-reported weight and BMI (248/250) ($Ps > 0.11$). At 12-weeks among participants with a measured weight (124/250), average self-reported weight was 0.7 kg (± 1.3 SD) less than the measured weight ($P < 0.001$). However, there was no difference between intervention (56/125; 0.8 kg ± 1.2 SD) and control groups (68/125; 0.6 kg ± 1.4 SD) ($P = 0.44$). There was no difference between measured and self-reported BMI at 12-weeks ($P = 0.26$). "

18-i) Subgroup analysis of comparing only users

A subgroup analysis of comparing only users is not uncommon in ehealth trials, but if done, it must be stressed that this is a self-selected sample and no longer an unbiased sample from a randomized trial (see 16-iii).

	1	2	3	4	5	
subitem not at all important	○	○	●	○	○	essential

Does your paper address subitem 18-i?

Copy and paste relevant sections from the manuscript (include quotes in quotation marks "like this" to indicate direct quotes from your manuscript), or elaborate on this item by providing additional information not in the ms, or briefly explain why the item is not applicable/relevant for your study

Not applicable.

19) All important harms or unintended effects in each group

(for specific guidance see CONSORT for harms)

Does your paper address CONSORT subitem 19? *

Copy and paste relevant sections from the manuscript (include quotes in quotation marks "like this" to indicate direct quotes from your manuscript), or elaborate on this item by providing additional information not in the ms, or briefly explain why the item is not applicable/relevant for your study

No applicable. No harms or unintended effects in each group.

19-i) Include privacy breaches, technical problems

Include privacy breaches, technical problems. This does not only include physical “harm” to participants, but also incidents such as perceived or real privacy breaches [1], technical problems, and other unexpected/unintended incidents. “Unintended effects” also includes unintended positive effects [2].

	1	2	3	4	5	
subitem not at all important	○	○	○	○	●	essential

Does your paper address subitem 19-i?

Copy and paste relevant sections from the manuscript (include quotes in quotation marks "like this" to indicate direct quotes from your manuscript), or elaborate on this item by providing additional information not in the ms, or briefly explain why the item is not applicable/relevant for your study

No applicable. No privacy breaches, technical problems.

19-ii) Include qualitative feedback from participants or observations from staff/researchers

Include qualitative feedback from participants or observations from staff/researchers, if available, on strengths and shortcomings of the application, especially if they point to unintended/unexpected effects or uses. This includes (if available) reasons for why people did or did not use the application as intended by the developers.

	1	2	3	4	5	
subitem not at all important	○	○	○	○	●	essential

Does your paper address subitem 19-ii?

Copy and paste relevant sections from the manuscript (include quotes in quotation marks "like this" to indicate direct quotes from your manuscript), or elaborate on this item by providing additional information not in the ms, or briefly explain why the item is not applicable/relevant for your study

Qualitative process evaluation currently in progress and will form the basis of another manuscript.

DISCUSSION

22) Interpretation consistent with results, balancing benefits and harms, and considering other relevant evidence

NPT: In addition, take into account the choice of the comparator, lack of or partial blinding, and unequal expertise of care providers or centers in each group

22-i) Restate study questions and summarize the answers suggested by the data, starting with primary outcomes and process outcomes (use)

Restate study questions and summarize the answers suggested by the data, starting with primary outcomes and process outcomes (use).

	1	2	3	4	5	
subitem not at all important	○	○	○	○	◉	essential

Does your paper address subitem 22-i? *

Copy and paste relevant sections from the manuscript (include quotes in quotation marks "like this" to indicate direct quotes from your manuscript), or elaborate on this item by providing additional information not in the ms, or briefly explain why the item is not applicable/relevant for your study

In discussion section: "This 12-week TXT2BFiT m-Health intervention was effective in preventing unhealthy weight gain resulting in modest weight loss and improvement in lifestyle behaviours. Compared with control participants, intervention participants reported consuming more vegetables, and less SSB, consumed energy-dense meals prepared away from home less often, and increased their physical activity, with increased total and walking days of physical activity. As far as we are aware this is the first reported trial of a multi-component mobile phone based program conducted in young adults."

22-ii) Highlight unanswered new questions, suggest future research

Highlight unanswered new questions, suggest future research.

	1	2	3	4	5	
subitem not at all important	○	○	○	○	◉	essential

Does your paper address subitem 22-ii?

Copy and paste relevant sections from the manuscript (include quotes in quotation marks "like this" to indicate direct quotes from your manuscript), or elaborate on this item by providing additional information not in the ms, or briefly explain why the item is not applicable/relevant for your study

In discussion section: "While the short-term efficacy of the TXT2BFiT intervention program is promising, maintenance of outcomes longer term require evaluation. The potentially wide reach and low delivery costs of using m-Health, coupled with the growing problem of obesity in younger adulthood, means translation and implementation of this program to the community-at-large also warrants further consideration."

20) Trial limitations, addressing sources of potential bias, imprecision, and, if relevant, multiplicity of analyses

20-i) Typical limitations in ehealth trials

Typical limitations in ehealth trials: Participants in ehealth trials are rarely blinded. Ehealth trials often look at a multiplicity of outcomes, increasing risk for a Type I error. Discuss biases due to non-use of the intervention/usability issues, biases through informed consent procedures, unexpected events.

	1	2	3	4	5	
subitem not at all important	○	○	○	○	●	essential

Does your paper address subitem 20-i? *

Copy and paste relevant sections from the manuscript (include quotes in quotation marks "like this" to indicate direct quotes from your manuscript), or elaborate on this item by providing additional information not in the ms, or briefly explain why the item is not applicable/relevant for your study

In discussion section: "The use of GP's to measure height and weight on their scales could introduce measurement error, but GP clinic was one of the strata for participant randomization and observer and equipment error would have been distributed equally across groups and this should not have impacted the results from the analysis of covariance. As self-reported measures were used for all studied outcomes, the data may be biased. Self-report may underestimate weight, but has shown to accurately identify overweight and/or obesity in the majority of a sample of young people [46]. An element of social desirability might influence reporting of lifestyle behaviours. Both groups were provided dietary and physical activity guidelines; however, greater significant improvements in intervention participants were seen in this study. Further, the sample was mostly well educated and from higher socioeconomic areas, which may influence the generalizability of the results [47]."

21) Generalisability (external validity, applicability) of

the trial findings

NPT: External validity of the trial findings according to the intervention, comparators, patients, and care providers or centers involved in the trial

21-i) Generalizability to other populations

Generalizability to other populations: In particular, discuss generalizability to a general Internet population, outside of a RCT setting, and general patient population, including applicability of the study results for other organizations

	1	2	3	4	5	
subitem not at all important	○	○	○	○	◉	essential

Does your paper address subitem 21-i?

Copy and paste relevant sections from the manuscript (include quotes in quotation marks "like this" to indicate direct quotes from your manuscript), or elaborate on this item by providing additional information not in the ms, or briefly explain why the item is not applicable/relevant for your study

In discussion section: "Further, the sample was mostly well educated and from higher socioeconomic areas, which may influence the generalizability of the results [47]."

21-ii) Discuss if there were elements in the RCT that would be different in a routine application setting

Discuss if there were elements in the RCT that would be different in a routine application setting (e.g., prompts/reminders, more human involvement, training sessions or other co-interventions) and what impact the omission of these elements could have on use, adoption, or outcomes if the intervention is applied outside of a RCT setting.

	1	2	3	4	5	
subitem not at all important	○	○	○	○	◉	essential

Does your paper address subitem 21-ii?

Copy and paste relevant sections from the manuscript (include quotes in quotation marks "like this" to indicate direct quotes from your manuscript), or elaborate on this item by providing additional information not in the ms, or briefly explain why the item is not applicable/relevant for your study

Process evaluation is currently in progress.

OTHER INFORMATION

23) Registration number and name of trial registry

Does your paper address CONSORT subitem 23? *

Copy and paste relevant sections from the manuscript (include quotes in quotation marks "like this" to indicate direct quotes from your manuscript), or elaborate on this item by providing additional information not in the ms, or briefly explain why the item is not applicable/relevant for your study

In abstract section: "The trial is registered with the Australian New Zealand Clinical Trials Registry (ACTRN 12612000924853)."

24) Where the full trial protocol can be accessed, if available

Does your paper address CONSORT subitem 24? *

Cite a Multimedia Appendix, other reference, or copy and paste relevant sections from the manuscript (include quotes in quotation marks "like this" to indicate direct quotes from your manuscript), or elaborate on this item by providing additional information not in the ms, or briefly explain why the item is not applicable/relevant for your study

In methods section: "Both the protocol and recruitment methods have been previously published [22]."

25) Sources of funding and other support (such as supply of drugs), role of funders

Does your paper address CONSORT subitem 25? *

Copy and paste relevant sections from the manuscript (include quotes in quotation marks "like this" to indicate direct quotes from your manuscript), or elaborate on this item by providing additional information not in the ms, or briefly explain why the item is not applicable/relevant for your study

Under sources of funding "Funding to conduct this trial was received from the HCF Medical Research Foundation (Reference number MAUsyd1008201111), listed on the Australian Competitive Research Grants Register Category 1. This work was also supported by the Commonwealth Government of Australia via an Australian Postgraduate Award Scholarship to SRP and LH was supported by a National Health and Medical Research Council Scholarship between 2011 and 2013. MFH is partly funded by a NHMRC Senior Research [illegible]

X27) Conflicts of Interest (not a CONSORT item)

X27-i) State the relation of the study team towards the system being evaluated

In addition to the usual declaration of interests (financial or otherwise), also state the relation of the study team towards the system being evaluated, i.e., state if the authors/evaluators are distinct from or identical with the developers/sponsors of the intervention.

	1	2	3	4	5	
subitem not at all important	○	○	○	○	◉	essential

Does your paper address subitem X27-i?

Copy and paste relevant sections from the manuscript (include quotes in quotation marks "like this" to indicate direct quotes from your manuscript), or elaborate on this item by providing additional information not in the ms, or briefly explain why the item is not applicable/relevant for your study

Under competing interests: "No competing interests."

About the CONSORT EHEALTH checklist

As a result of using this checklist, did you make changes in your manuscript? *

- ○ yes, major changes
- ◉ yes, minor changes
- ○ no

What were the most important changes you made as a result of using this checklist?

Small details necessary to improve the external validity reporting.

How much time did you spend on going through the checklist INCLUDING making changes in your manuscript *

5 hours

As a result of using this checklist, do you think your manuscript has improved? *

- (•) yes
- ( ) no
- ( ) Other:

Would you like to become involved in the CONSORT EHEALTH group?

This would involve for example becoming involved in participating in a workshop and writing an "Explanation and Elaboration" document

- ( ) yes
- (•) no
- ( ) Other:

Any other comments or questions on CONSORT EHEALTH

No.

STOP - Save this form as PDF before you click submit

To generate a record that you filled in this form, we recommend to generate a PDF of this page (on a Mac, simply select "print" and then select "print as PDF") before you submit it.

When you submit your (revised) paper to JMIR, please upload the PDF as supplementary file.

Don't worry if some text in the textboxes is cut off, as we still have the complete information in our database. Thank you!

Final step: Click submit !

Click submit so we have your answers in our database!

Submit

Never submit passwords through Google Forms.

Powered by

This content is neither created nor endorsed by Google.

Report Abuse - Terms of Service - Additional Terms
